# Stereotypic wheel running decreases cortical activity in mice

**DOI:** 10.1038/ncomms13138

**Published:** 2016-10-17

**Authors:** Simon P. Fisher, Nanyi Cui, Laura E. McKillop, Jessica Gemignani, David M. Bannerman, Peter L. Oliver, Stuart N. Peirson, Vladyslav V. Vyazovskiy

**Affiliations:** 1 Department of Physiology, Anatomy and Genetics, University of Oxford, Parks Road, Oxford OX1 3PT, UK; 2 European Space Agency, Advanced Concepts Team, Keplerlaan 1, 2201 Noordwijk, The Netherlands; 3 Department of Experimental Psychology, University of Oxford, South Parks Road, Oxford OX1 3UD, UK; 4 Sleep and Circadian Neuroscience Institute, Nuffield Department of Clinical Neurosciences, University of Oxford, South Parks Road, Oxford OX1 3RE, UK

## Abstract

Prolonged wakefulness is thought to gradually increase ‘sleep need' and influence subsequent sleep duration and intensity, but the role of specific waking behaviours remains unclear. Here we report the effect of voluntary wheel running during wakefulness on neuronal activity in the motor and somatosensory cortex in mice. We find that stereotypic wheel running is associated with a substantial reduction in firing rates among a large subpopulation of cortical neurons, especially at high speeds. Wheel running also has longer-term effects on spiking activity across periods of wakefulness. Specifically, cortical firing rates are significantly higher towards the end of a spontaneous prolonged waking period. However, this increase is abolished when wakefulness is dominated by running wheel activity. These findings indicate that wake-related changes in firing rates are determined not only by wake duration, but also by specific waking behaviours.

## Abstract (web summary)

Sleep need is thought to accumulate gradually over waking periods and is associated with changes in neuronal activity. Here the authors show that in mice cortical firing rates increase between the beginning and end of wakefulness periods but this increase is not seen in waking periods with voluntary stereotypic wheel running.

## Graphical abstract

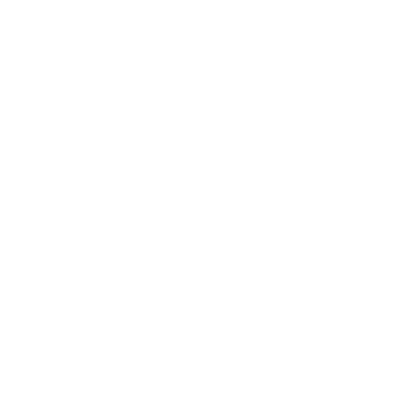

Global sleep-wake switching is controlled through a dynamic interplay within several subcortical areas, which integrate external and internal information including the time of day, metabolic requirements and immediate and long term preceding history of activity^1^,^2^. The concept of sleep homeostasis refers to the maintenance of a balance between waking and sleep on a time scale of ∼24 h (ref. 3). Following prolonged wakefulness, non-rapid eye movement (NREM) sleep is typically enhanced, and is characterized by increased levels of electroencephalogram (EEG) slow wave activity (0.5–4 Hz), which is considered a measure of sleep ‘intensity'. It is posited that longer and more ‘intense' sleep compensates for sleep loss, and thus the balance is restored. The nature of the mechanism that tracks time spent awake (and hence sleep need) remains elusive; particularly, because wakefulness is a highly dynamic state, as manifested in continual changes of cortical activity as a function of ongoing behaviour, attention and sensory input^4^. Behavioural state-dependency of neuronal activity is typically cortical region- or layer-specific and often pertains to highly idiosyncratic and dynamic changes among specific populations of cortical neurons^5^,^6^,^7^,^8^,^9^,^10^,^11^,^12^,^13^,^14^.

The well-known variability and diversity of spontaneous behaviours in freely moving animals is likely to represent an important variable for our understanding of the nature of the wake-dependent increase in sleep need. One possibility is that sleep pressure builds up during waking irrespective of ongoing behaviour, as long as the animal remains awake. However, it seems likely that the nature and complexity of waking behaviours have unique and distinct contributions to the dynamics of sleep pressure accumulation. It has been suggested that sleep has properties of an activity-dependent process, whereby it is initiated at the level of local neuronal networks, which were more active during prior waking, and then progressively encompasses larger areas of the brain, before eventually precipitating global behavioural sleep^15^,^16^,^17^. Consistently, it has been shown that the amount of exploratory behaviour during sleep deprivation, or the nature of the procedure employed to keep animals awake, can affect subsequent sleep latency and EEG slow wave activity^18^,^19^. While the precise underlying mechanisms are unclear, it is possible that the levels of spiking or synaptic activity during waking influence the ongoing dynamics of the sleep homeostatic process. Although it has been suggested that being awake or asleep may alter the directionality of the changes in synaptic strength, the view that these two states have opposing effects on neuronal excitability or firing rates has so far yielded conflicting evidence^20^,^21^,^22^,^23^,^24^,^25^,^26^, and the role of behaviour and brain state in the dynamics of this process remains unclear.

An intriguing possibility, which has not been tested previously, is that waking behaviours associated with low or high network neuronal activity have a differential effect on the rate of accumulation of ‘global' sleep need across time spent awake. In this study, we performed continuous recordings of neuronal activity from two cortical regions in mice given free access to running wheels (RWs). Previously, it has been shown that spontaneous wheel running is associated with distinct changes in the architecture and distribution of vigilance states across 24 h, and leads to regional changes in waking and sleep EEG^27^,^28^,^29^,^30^. On the other hand, several studies performed in head-fixed animals showed that locomotion affects neuronal activity in a cortical region, and cell type-specific manner^6^,^7^,^8^,^9^,^13^. Little is known, however, about the effects of prolonged periods of stereotypic running on cortical activity in freely moving animals. In this study, we have investigated whether wheel running behaviour in freely moving, non-head-fixed mice, is associated with changes in firing rates in the primary motor cortex (M1) and the somatosensory cortex (SCx), both in relation to running characteristics per se, as well as with respect to dynamic changes in cortical activity across continuous periods of spontaneous wakefulness. We found that wheel running was associated with longer periods of sustained wakefulness, and cortical neuronal activity in M1 and SCx was reduced during stereotypic high speed running. Overall firing rates in these two cortical areas increased as a function of wake duration, but this increase was abolished during waking periods dominated by RW activity.

## Results

### Cortical neuronal activity during wheel running

**Figure 1:**
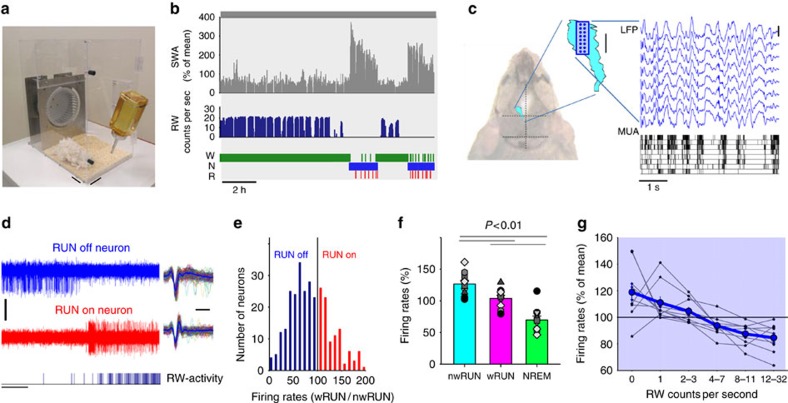
Cortical neuronal activity in the primary motor cortex (M1) during voluntary wheel running in freely behaving mice. (**a**) A photograph of the custom-made cage providing continuous free access to a running wheel positioned at the rear. Scale bars, 5 cm. (**b**) From top to bottom: 12-h profile of EEG slow-wave activity (SWA, EEG power between 0.5–4.0 Hz, represented as % of 12-h mean) recorded in the frontal cortex, running-wheel (RW) activity (counts per second) and the distribution of sleep-wake stages (W=wakefulness, N=NREM sleep, R=REM sleep) from a representative mouse. (**c**) Schematic representation of the position of the primary motor cortex (M1, blue area drawn with reference to Paxinos & Franklin, 2001) shown on the dorsal surface of the mouse head, and the position of the 16-ch microwire array above M1 (dots indicate the position of individual wires within the array, scale bar, 1 mm). Traces on the right: top, representative local field potentials (LFP) recorded during NREM sleep from one row of microwires; bottom, raster plot of multiunit activity (MUA, each vertical line represents a spike) recorded from the same wires (scale bar, 500 μV). Note the close temporal relationship between positive LFP waves and periods of generalized neuronal silence. (**d**) Two MUA traces recorded from M1 in the same animal with corresponding RW-activity (bottom, each vertical bar represents a single wheel rung count, scale bars, amplitude 100 μV, time 1 s). Corresponding waveforms of the action potentials recorded extracellularly are shown on the right (scale bar, 0.5 ms). (**e**) The distribution of all putative single units recorded in *n*=11 mice as a function of the ratio of their average firing rates (FR) during running (wRUN) and non-running waking (nwRUN). Note that a smaller proportion of neurons increase firing during wheel running (red, RUN on neurons), while the majority decrease FR during running (blue, RUN off neurons). (**f**) Average FR in M1 during nwRUN waking, wRUN waking and NREM sleep. Mean values, s.e.m., *n*=11 (individual mice: grey symbols). Significant differences between vigilance states are depicted above the bars (obtained by two-tailed paired *t*-test following significant one-way ANOVA). (**g**) FR in M1 shown as function of running speed. Thick line: mean values, s.e.m., *n*=9 mice. Values from individual animals are shown as thin line plots.

In the first experiment, multiunit activity (MUA) in the motor cortex and EEG were recorded in mice which had free access to a RW in their home cage (Fig. 1a, Supplementary Fig. 1). Before the experiment, the animals were habituated to the wheel for 15.3±2.1 days and ran spontaneously on average more than 800 meters within the 12 h experimental dark period (868.7±161 m, Supplementary Fig. 2). In every animal, several prolonged waking periods dominated by RW activity were observed during the dark period, interspersed by naps of various durations (Fig. 1b). As typically observed^30^, during waking epochs with wheel running (wRUN), EEG was dominated by theta-activity (6–9 Hz), which was substantially lower during waking epochs when the animals did not run (nwRUN, Supplementary Fig. 2b). We also observed that during NREM sleep the cortical local field potential (LFP) recorded from M1 was dominated by high-amplitude positive slow waves (∼0.5–4 Hz) associated with a brief reduction of MUA across the majority or even all of the electrodes within the 16-channel array (Fig. 1c).

In contrast, during waking we observed a striking dissociation between the activities of putative single units depending on the animal's behaviour (Fig. 1d). Specifically, in every mouse we observed clear cases where MUA slowed down substantially or ceased altogether during intense running on the wheel (RUN off neurons, Fig. 1d,e, Supplementary Movies 1–2). Overall, cortical neuronal activity was on average 25.0±9.3% lower during wRUN compared with nwRUN waking (repeated measures analysis of variance (ANOVA), factor ‘vigilance/behavioural state', F(2,18)=17,23, *P*=0.0001, Fig. 1f). We should emphasize, that although RUN off neurons comprised a larger population of recorded neurons, and overall cortical activity on average decreased during running, individual putative single units were highly variable with respect to their relationship with running (Fig. 1d,e), and neurons which increased firing during running (RUN on neurons) were not uncommon (Supplementary Movie 2). Moreover, most neurons did not merely change firing when the animals ran, but they often showed a distinct inverse relationship to running velocity (Supplementary Fig. 3). This resulted in a significant negative relationship between the firing rates in M1 and running speed (Fig. 1g. F(5,53)=8,61, *P*=1.3315e-005, ANOVA for repeated measures, factor ‘running speed').

It is possible that the differential involvement of neurons in running or non-running behaviour is determined by their specific topographic location. Since the microwire arrays used to record MUA in this study were 1,750 μm long in the antero-posterior dimension (Fig. 1c), they span various parts of the motor cortex topographically representing different body parts^31^. To address this, we grouped the 16 wire array electrodes into four clusters (each consisting of four wire electrodes) along the antero-posterior axis (Supplementary Fig. 4), and calculated the average firing rates, as well as the proportion of RUN off neurons for each of the four clusters of electrodes independently. Interestingly, a higher proportion of RUN off neurons were recorded from the anterior electrodes of the array (F(3,35)=3.04, *P*=0.049, ANOVA for repeated measures, factor ‘anterior-posterior gradient'), although firing rates were on average similarly decreased across the entire M1 array during wRUN waking compared with nwRUN waking (F(3,35)=1.27, *P*=0.31).

**Figure 2:**
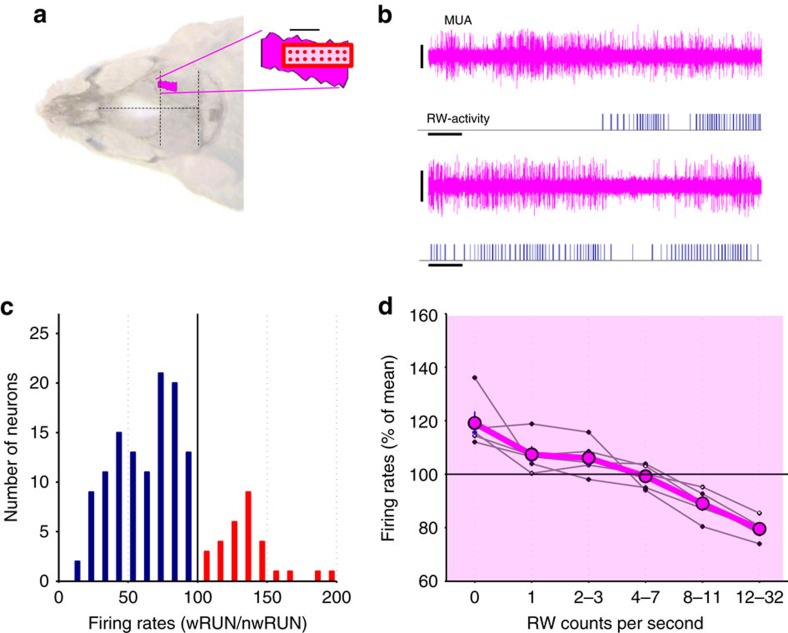
Cortical neuronal activity in the somatosensory cortex (SCx) during voluntary wheel running. (**a**) Schematic representation of the position of the 16-ch microwire array shown relative to the barrel cortex (drawn in reference to Paxinos & Franklin, 2001) shown on the dorsal surface of the mouse head (dots indicate the position of individual wires within the array, scale bar, 1 mm). (**b**) Individual representative examples depicting multiunit activity (MUA) in SCx during wheel running (scale bars: amplitude 100 μV, time 1 s). Corresponding running wheel (RW)-activity is shown below each trace (each vertical bar represents a single wheel rung count). (**c**) The distribution of all putative single units recorded in SCx (in *n*=5 mice) as a function of the ratio of their average firing rates (FR) during running (wRUN) and non-running waking (nwRUN). Note that a smaller proportion of neurons increase FR during running (red), while the majority decrease spiking activity (blue). (**d**) FR in SCx shown as a function of running speed (counts per second). Thick line: mean values, s.e.m., *n*=5 mice. Values from individual animals are shown as thin line plots.

To investigate whether spontaneous wheel running affects firing rates in other cortical areas, we also performed MUA recordings from the SCx (Fig. 2). Although visual inspection did not reveal individual neurons in SCx that would stop firing altogether during running as observed in M1 (Supplementary Movie 2), the majority of neurons also changed their firing activity during running. Again, we observed substantial variability between individual neurons with respect to their relationship with running behaviour, similar to observations in M1 (Supplementary Fig. 5). On average, firing rates in the SCx also showed a decrease with increasing running speed (Fig. 2, F(5,29)=19,42, *P*=4.6534e-007, ANOVA for repeated measures, factor ‘running speed'), although in contrast to the M1, the position of the wire within the 16-channel array had a noticeable effect. Specifically, the difference in firing rates between wRUN and nwRUN waking was more pronounced among neurons recorded by more anterior electrodes of the array (F(3,19)=9.21, *P*=0.002, ANOVA for repeated measures, factor ‘anterior-posterior gradient', Supplementary Fig. 4), although the proportion of RUN off neurons was high and relatively uniform throughout the entire SCx array (F(3,19)=1.31, *P*=0.35).

Thus, we observed a relatively consistent reduction in average spiking activity during running in two cortical regions. On the other hand, it was apparent that in each of these regions individual neurons were highly variable with respect to the change in their firing activity during running (Figs 1, 2 and Supplementary Fig. 6a). Notably, the average firing rates during nwRUN waking were similar between those neurons, which were found to increase or decrease spiking during running (Supplementary Fig. 6b). Furthermore, a correlation analysis between firing rates and the ratio of spiking frequency between wRUN and nwRUN waking failed to detect a significant relationship (Supplementary Fig. 6c), suggesting that average firing rates are not informative of the RUN on / RUN off phenotype. Finally, only a weak negative correlation emerged between spike width and the ratio of firing rates between wRUN/nwRUN waking, which reached statistical significance for putative single units recorded in M1 only (Supplementary Fig. 6d–e).

### Stereotypic running decreases cortical firing rates

**Figure 3:**
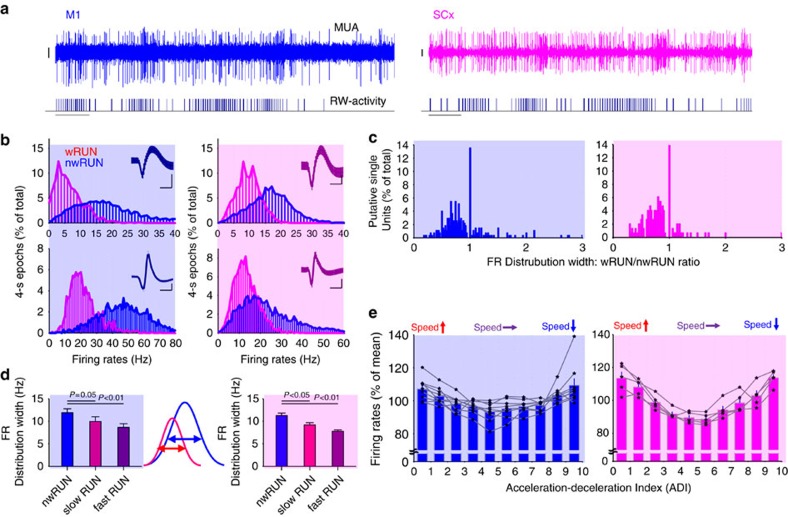
The relationship between cortical neuronal activity and changes in wheel running speed. (**a**) Individual examples depicting cortical firing rates (FR) in the motor cortex (M1) and somatosensory cortex (SCx) modulated by running wheel behaviour. From top to bottom: MUA recorded in one representative channel of the 16-channel array, and running-wheel (RW)-activity (each vertical bar represents a single wheel rung count, scale bars, amplitude 50 μV, time 1 s). Note the irregular pattern of spiking associated with variability in running speed. (**b**) The distribution of 1-s epochs as a function of firing rates during wRUN and nwRUN waking. Four individual putative single units are shown. Blue background relates to data recorded in M1, pink background relates to data recorded in SCx. Note that running is associated with a substantial shift in the distribution of firing rates. Insets: corresponding spike wave forms (scale bars, amplitude 50 μV, time 0.5 ms). (**c**) The distribution of all putative single units recorded in M1 (*n*=9 mice) and SCx (*n*=5 mice) as a function of the ratio of the width of FR distribution during wRUN and nwRUN waking. Note that the majority of neurons have narrower distribution of FRs during wRUN waking in both M1 and SCx. (**d**) The effect of wheel running speed on the width of FRs distribution. Left and right panels: mean values for M1 and SCx, respectively. The curves in the middle schematically depict the procedure for calculating the width of FRs distributions at ½ of their height. Values above depict *P* values of paired *t*-test (after Bonferroni's correction). (**e**) Cortical FR in M1 (blue) and SCx (pink) shown as a function of the wheel acceleration–deceleration index. All 1-s epochs were subdivided into ten 10% deciles, each consisting of the same number of epochs. These were sorted as a function of the change in wheel running speed within an epoch from fast acceleration to fast deceleration (schematically shown as arrows above), and corresponding average FR were calculated before averaging between animals. Mean values, s.e.m., M1: *n*=9 mice, SCx: *n*=5. Mean values are shown as bar plots, the individual values from individual animals are shown as thin line plots.

Spontaneous wheel running is not stable and uniform throughout running bouts. Instead we frequently observed brief pauses in running, and fluctuations in running speed were common, irrespective of the running bout length. On the other hand, moment-to-moment changes in firing rates were frequently observed both within wRUN and nwRUN waking (Fig. 3a,b). Most putative single units had a ‘preferred' frequency of firing, which was affected substantially by wheel running behaviour (Fig. 3b). However, we noticed that the width of the distribution of firing rates was substantially reduced when the mice engaged in running, especially at a high speed, an effect which was present among the majority of individual putative single units (Fig. 3c). This suggests that during running a more uniform or stereotyped cortical state is instated, which contrasted significantly with the wider dynamic range of firing during nwRUN waking (Fig. 3d; M1: F(4,44)=93.86, *P*<0.0001, SCx: F(4,24)=278.88, *P*<0.0001, ANOVA for repeated measures, factor ‘RW-activity').

Striking changes in the firing activity depending on the pattern and nature of running behaviour, such as during initial acceleration, during steady constant running and during brief pauses in RW-activity were also observed (Supplementary Movies 3–4). To further investigate the relationship between the mode of wheel running and firing rates, we calculated a wheel acceleration/deceleration index (ADI) based upon the timing of individual RW counts during 1-s epochs. For most epochs the average ADI was close to 0 representing negligible net speed changes within an epoch, as typical for stereotypic steady speed running. Although abrupt fluctuations in running speed, corresponding to positive or negative ADI values during a consolidated running bout were also encountered (Supplementary Fig. 7). Firing rates were strongly related to the ADI in both M1 and SCx (M1: F(9,89)=6.31, *P*=1.5427e-006, SCx: F(9,49)=13,9, *P*=2.5696e-009). Specifically, firing rates were on average 20–30% lower during epochs with steady running, as compared with epochs with rapid acceleration or deceleration (Fig. 3e). Calculating the relationship between firing rates and ADI separately for RUN on and RUN off neurons revealed overall lower firing rates during steady running irrespective of whether the neuron increased or decreased its firing rate relative to nwRUN waking in both regions (Supplementary Fig. 8). In other words, even if a specific neuron fired more strongly during wRUN waking as compared with nwRUN waking, it tended to fire less frequently as running became more stereotypic.

Since mice use wheels spontaneously during the night, the question remains whether the relationship between cortical activity and running behaviour is similar at different times of day, under different lighting conditions, and when they are given a choice of other behaviours. To address this, we analysed data collected during the light period, when the animals were kept awake for 6 h, by continuously providing them with novel objects in their home cages (sleep deprivation protocol; Supplementary Fig. 9). As expected, the mice spent time exploring the novel objects, but they would also often run on the wheel, running on average ∼200 meters during a 6-h sleep deprivation protocol. Running was distributed evenly across the period of sleep deprivation (F(5,53)=1.24, *P*=0.31, ANOVA for repeated measures, factor ‘1-h interval', Supplementary Fig. 9a,b), suggesting that increased sleep pressure did not prevent the mice from running, even at a time of day when they typically do not run. Notably, in all individual animals we again observed clear cut cases of putative single units decreasing spiking substantially during running (Supplementary Fig. 9). In all animals, firing rates also decreased with increasing running speed during sleep deprivation, similar to spontaneous running during the dark phase (Supplementary Fig. 9d).

**Figure 4:**
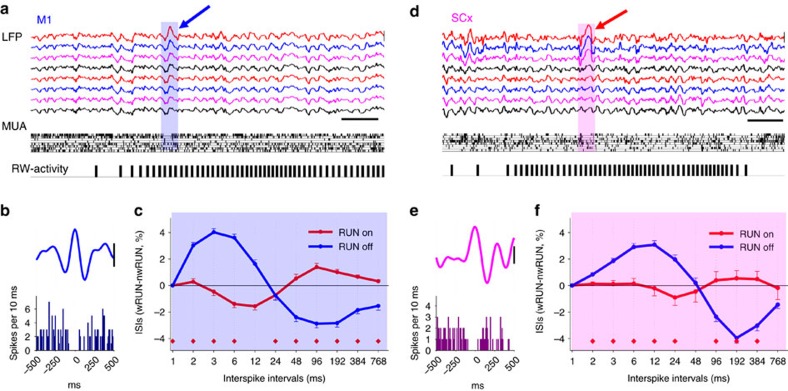
Cortical activity during voluntary wheel running. (**a**) Top traces show representative local field potentials (LFP, 0.1–100 Hz) in eight channels of the 16-channel microwire array placed in the primary motor cortex (M1). Middle: raster plot of multiunit activity (MUA) recorded in the same eight channels (each vertical line represents a spike). Bottom: corresponding running-wheel (RW)-activity (each bar represents a single wheel rung count). Note that a positive LFP wave is accompanied by reduced MUA in corresponding channels (scale bars, amplitude 500 μV, time 0.5 s). (**b**) Individual representative example of a positive LFP slow (2–6 Hz) wave recorded with one individual wire of a 16-channel microwire array during wheel running. Bar plot below shows corresponding MUA (scale-bar: 200 μV). (**c**) The distribution of interspike intervals (ISIs) as a function of wheel running behaviour. The number of ISIs calculated as a function of logarithmically increasing ISI duration bins, plotted against their lower limits. For each putative single unit ISIs were calculated during wRUN and nwRUN waking, and the difference between the two states was calculated separately for RUN on and RUN off neurons. Mean values, s.e.m., *n*=9 mice (red diamonds, *P*<0.05, paired *t*-test). Note a relative predominance of short ISIs during running among RUN off neurons, compared with nwRUN waking and RUN on neurons. (**d**,**e**,**f**) The same analysis for SCx. Mean values, s.e.m. (*n*=5 mice).

It is well established that state-dependent changes in cortical activity are accounted for by a variety of mechanisms, including intrinsic properties of individual neurons, local and global neuromodulation, active inhibition and disfacilitation, as well as connectivity within the network^32^,^33^,^34^,^35^,^36^. As all these factors may contribute to some extent to the effects we observed, going beyond merely calculating average firing rates may provide important insights. Visual inspection of raw traces revealed the occurrence of isolated brief (∼100–200 ms) periods of reduced neuronal spiking that corresponded to positive, slow fluctuations in the LFP, even during intense wheel running. Such events were encountered both in M1 and SCx (Fig. 4a,b,d,e, Supplementary Movie 5, Supplementary Fig. 9e), and usually encompassed a subset of recording channels, but in some cases were visible across the entire 16-channel array (Supplementary Fig. 10). Interestingly, these events were often characterized by a conspicuous brief surge of intense spiking activity immediately preceding and/or following the period of reduced firing (Supplementary Movie 5).

Next, we hypothesized that since individual neurons are highly variable with respect to the modulation of their average firing rates by running behaviour (Supplementary Fig. 6), the pattern of their activity may also be different. Therefore, we calculated the distribution of interspike intervals (ISIs) during wRUN and nwRUN waking for each putative single unit. Interestingly, in both M1 and SCx, shorter ISIs were more predominant among RUN off neurons (Fig. 4c,f), while RUN on neurons showed a higher frequency of longer ISIs in comparison to RUN off neurons, similar to those occurring during NREM sleep^20^.

### Running prevents wake-dependent increase in firing rates

**Figure 5:**
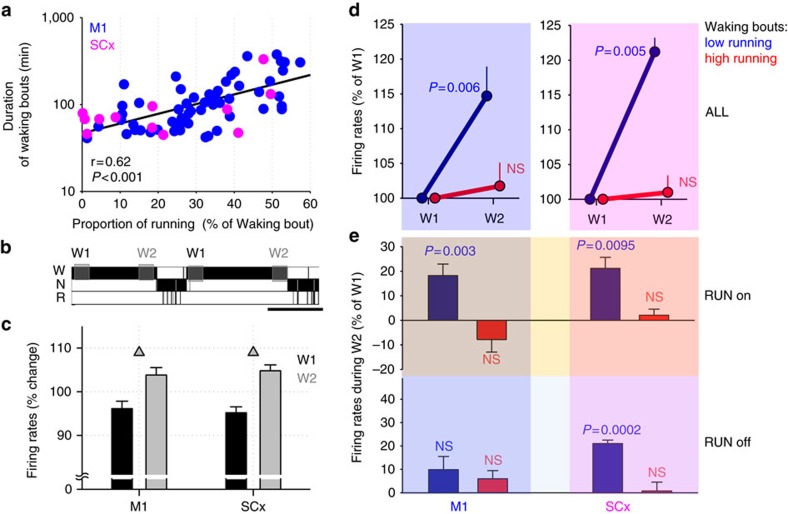
Longer-term effects of wheel running on cortical firing rates. (**a**) The relationship between the proportion of time spent running during a period of spontaneous waking and the duration of the corresponding waking period. Each dot corresponds to an individual waking period. The data are shown separately from animals implanted in M1 (*n*=11) and SCx (*n*=5). Note that a higher proportion of running is associated with longer waking periods. (**b**) Sleep-wake stages (W=wakefulness, N=NREM sleep, R=REM sleep) from one representative mouse showing two consolidated spontaneous waking periods. Shaded areas indicate the 15-min time intervals used for comparison between the beginning (W1) and end (W2) of waking periods >40 min. Scale bar, 1 h. (**c**) Average firing rates (FR) during W1 and W2 represented as % of the mean value between W1 and W2 for each putative single unit. Note that on average spontaneous cortical FR increased significantly (triangles, *P*<0.05, paired *t*-test) from W1 to W2 in both M1 and SCx. Mean values, s.e.m. (*n*=11 and *n*=5 animals for M1 and SCx, respectively). (**d**) Average FR during W1 and W2 shown separately for waking periods with a high or low amount of running (top and bottom 50% of the distribution of all waking periods, respectively = ‘high'—red, and ‘low'—blue). FR during W2 are shown as % of corresponding values during W1. Note that FR increased significantly only in the course of waking periods with low RW-activity. (**e**) Average FR during W2 shown as % of W1 shown separately for RUN on neurons (top) and RUN off neurons (bottom). As in **c**, blue bars correspond to waking periods with low RW-activity, and red bars correspond to waking periods with high RW-activity. NS, not significant.

As previously noted^30^, we observed that the amount of running during specific waking bouts was associated with the bout length, such that sustained waking bouts were longer when animals engaged in running behaviour (Fig. 5a). This observation led us to hypothesize that running not only results in instantaneous changes in cortical activity as a function of running speed, but also has long-term effects on cortical activity. To address this possibility, we first identified spontaneous waking periods lasting >40 min, and compared average firing rates across all recorded putative single units in the first 15 min (W1) and the last 15 min (W2) of each period (Fig. 5b, Supplementary Fig. 11). Average firing rates increased modestly (∼10%) from the beginning to the end of sustained waking periods, reaching statistical significance in both the M1 and SCx areas (Fig. 5c). We next subdivided all the waking periods into those with high and low amounts of RW-activity (top and bottom 50% of the distribution, with at least one waking period contributing to each category per animal, Supplementary Fig. 11), and calculated the corresponding changes in firing rates from the beginning to the end of each period (Fig. 5d). Importantly, the initial value at W1 did not differ significantly between waking periods with low and high RW-activity in M1 (‘high' running: 12.0±1.2 Hz, ‘low' running: 12.0±1.4 Hz, *P*=0.99), although in SCx the corresponding values were initially slightly lower during waking periods with low running (‘high' running: 9.6±0.7 Hz, ‘low' running: 9.0±0.8 Hz, *P*=0.08).

Notably, in both M1 and SCx an increase in neuronal firing from the beginning to the end of waking periods was only observed during those waking periods where the amount of RW-activity was low (M1: +14.7±4.2%, SCx: +21.2±2.0%, *P*<0.005, paired *t*-test, Fig. 5d), while the change was negligible during waking dominated by running behaviour (<2% in both M1 and SCx, Fig. 5d). This result suggests that the type of behaviour during a specific waking period contributes to wake-time-dependent changes in cortical firing rates. Although the selected waking periods with ‘high' and ‘low' amounts of running were not significantly different with respect to their duration (Supplementary Fig. 11), the possibility remains that the length of time the animals stayed awake is also important. To address this possibility, we next subdivided all waking periods based on their duration and compared the change of firing rates from W1 to W2 between the longest 50% and shortest 50% of waking periods. Interestingly, in this case, no significant change in firing rates was apparent (*P*=0.45 and 0.82 for M1 and SCx, respectively), suggesting that waking duration is less important than wake ‘quality', at least with respect to relatively short (∼2 h) spontaneous waking periods.

Next, we separated putative single units into those which fired, on average, at a relatively slow rate (<10 Hz) and those neurons, which discharged at a frequency above 10 Hz. In M1, a 15.4±5.2% increase in firing rate from W1 to W2 was found among ‘slow-spiking' units during waking bouts with ‘low running', which was not significantly different from a 12.0±3.1% increase observed among ‘fast-spiking' neurons (*P*=0.4). The corresponding values for waking bouts dominated by running were +1.3±5.3% and +4.5±3.4% for ‘slow-spiking' and ‘fast-spiking' units, respectively (*P*=0.31). Also in SCx, the increase from W1 to W2 during ‘low running' waking periods was similar between ‘slow-spiking' and ‘fast-spiking' putative single units (17.7±1.9 and 23.1±1.8, respectively, *P*=0.18), while firing rates remained stable in both groups of neurons when waking was dominated by running (−1.8±2.9 and +4.1±4.0, respectively, *P*=0.57). Finally, we calculated the difference in firing rates between the beginning and end of sustained waking periods separately for RUN on and RUN off neurons (Fig. 5e). Interestingly, these analyses yielded largely similar effects among RUN on and RUN off neurons, suggesting that the behaviour-dependent change in cortical excitability may be a widespread network phenomenon.

## Discussion

In this study, we report changes in the firing rates of cortical neurons recorded from motor (M1) and somatosensory (SCx) cortex in freely moving mice during voluntary unrestricted running-wheel (RW) activity. Importantly, we found that wheel running modulates a substantial proportion of cortical neurons, leading to an overall reduction in neuronal activity specifically during high speed and/or stereotypic running. Furthermore, our data suggest that changes in firing rates across prolonged periods of spontaneous wakefulness occur in a behaviour-dependent manner.

The mechanisms underlying the reduction in neuronal firing in M1 and SCx during high speed and/or stereotypic running may be diverse. As is well known, movement and behavioural state transitions are associated with widespread changes in the activity of several neuromodulatory systems^37^,^38^,^39^. Noradrenaline is essential for maintaining a tonic depolarization of pyramidal neurons in the motor, somatosensory and the visual cortex^8^,^40^,^41^. On the other hand, while wake-promoting effects of acetylcholine are well established^42^, *in vitro* studies suggest diverse layer-specific effects of acetylcholine, manifested in the hyperpolarization of layer four neurons and excitation of layer 2/3 and layer 5 pyramidal neurons^43^. In addition to ‘global' neuromodulatory influences extrinsic to the neocortex, local inhibition is also likely to be essential. Several studies have highlighted diversity among local GABA-ergic (γ-aminobutyric acid) neuronal populations that are recruited in awake mice in a unique manner with respect to ongoing behaviours, including movement or whisking^44^,^45^,^46^. Interestingly, pharmacogenetic inhibition of a subpopulation of vasoactive intestinal peptide positive (VIP) GABA-ergic neurons in the visual cortex resulted in reduced cortical activity irrespective of behavioural state^47^, while somatostatin-expressing GABA-ergic neurons of layer 2/3 of the barrel cortex were active during spontaneous quiet wakefulness, but reduced activity and hyperpolarized in response to whisking^48^. Moreover, further studies recording from the visual cortex showed that VIP interneurons were activated by locomotion, and in turn inhibited somatostatin-expressing interneurons, thus disinhibiting excitatory cells^49^. In turn, stimulation of excitatory neurons may lead to a strong activation of GABA-ergic neurons, which precipitates as a net inhibitory effect in the local microcircuit^50^. The observation of brief, slow LFP fluctuation events during running associated with a surge of neuronal spiking and subsequent MUA suppression, may reflect an arrival of extrinsic excitatory inputs recruiting local cortical inhibitory networks^51^,^52^. However, it remains to be established whether these slow LFP events, which we observed during intense running share common cellular and network mechanisms with slow waves occurring during NREM sleep.

In our study, average basal firing rates or spike wave-shape characteristics were poor predictors of the RUN on or RUN off phenotype. Instead, a strong determinant of the overall change in network activity was the pattern of running, such as whether it was stereotypic. Intriguingly, decreases in firing rate during stereotypic running were observed not only among RUN off neurons, but also among RUN on neurons, which on average fired at a higher rate when the animals ran, yet they were relatively quiescent as the animal engaged in a stable speed running. Notably, in our study the animals were habituated to spontaneous wheel running in their home cages and had access to the wheels for many days before the experimental night. It is possible that the effects we observed may have been different if the recordings had been obtained while the animals were learning how to run, as in this case the running would likely be less stereotypic, and instead may require more of an active involvement of the motor cortex^14^,^53^. It is also currently unknown whether the changes we observed in M1 and SCx during voluntary wheel running can be generalized to other cortical regions, such as the auditory or visual cortex. Several recent studies performed in head-fixed animals have reported a general suppression of firing rates in the auditory cortex during locomotion^13^, while enhancement of cortical activity was observed in the visual cortex, although variability between individual neurons has been noted^5^,^6^,^7^,^8^.

While our data indicates that stereotypic behaviours may translate into cortical states that are more homogenous and lack complexity, and in turn influence the dynamics of the accumulation of sleep need, the question remains whether other factors also have a role. Arguably, high-speed stereotypic running and goal-directed or exploratory behaviours are not only associated with a different type and amount of locomotion, but also with differences in the levels of arousal, which can in turn modulate sensory processing and behavioural performance^6^,^54^. On the other hand, it has been shown that prolonged wakefulness itself affects local cortical states and performance in behavioural tasks^15^,^55^, suggesting a complex relationship between preceding sleep-wake history, arousal levels and specific waking activities. Consistently, we observed that wheel running resulted not only in an instantaneous behaviour-dependent suppression of spiking across a population of cortical neurons, but also had pronounced long-term effects. Specifically, we found that the increase in spiking activity from the beginning to the end of spontaneous waking episodes was abolished when the wakefulness was dominated by running. The mechanisms underlying this effect remain to be determined, but could involve homeostatic plasticity^24^, increased inhibition among a subpopulation of neurons^33^,^56^, or altered levels of neuromodulators^57^. We would like to emphasize that it would be premature to conclude that the dynamic changes in firing rates observed here arise from changes in synaptic strength. As we clearly demonstrate in our study, ongoing behaviour has profound instantaneous effects on cortical firing rates, which are unique and diverse among individual neurons. We should point out, however, that we did not observe notable differences between RUN on and RUN off neurons or between ‘fast-spiking' and ‘slow-spiking' units with respect to their wake-time dependent changes in firing rates. This suggests that the changes we observed may be a generalized network phenomenon, representing a combined effect of mechanisms intrinsic and extrinsic to the neocortex.

The role of motivation in spontaneous wheel running also represents an important issue. Recent studies suggest that RW-activity resembles some forms of naturalistic behaviours^58^, and may have an intrinsic reinforcement value^59^. In rats, this was demonstrated using conditioned place preference, where prolonged access to the wheel was associated with plasticity in the mesolimbic reward circuitry, including the nucleus accumbens and the ventral tegmental area^60^. Consistent with this, it has recently been shown that mice have a preference towards wheel running when given a choice between engaging in this behaviour and eating a highly palatable food, an effect that was related to dopaminergic transmission^61^. To our surprise, in this study the mice readily ran during sleep deprivation performed during the light period, despite this being a habitual sleep period where mice typically do not show RW behaviour and are less active overall. Furthermore, during sleep deprivation the animals were constantly provided with novel objects, but rather than spending time exploring, the animals often chose to engage in stereotypical running. While the functional significance of this behaviour remains to be determined, our data suggest that even when wheel running occurs outside of the usual time and setting and under conditions of increased sleep pressure, the effects on cortical activity are similar to that observed during spontaneous wheel running in the dark phase.

The possibility remains that the total amount of intense locomotor activity, rather than the type of movement during running may be also essential in determining the dynamics of sleep need or the overall daily distribution of waking and sleep, and future studies are necessary to address this possibility. This aspect is particularly relevant, given that voluntary RW-activity is a widely used method to investigate circadian rhythms of behaviour in freely moving mice^62^. Intriguingly, our data suggest that the very assay that is used to measure spontaneous ‘activity' and ‘rest' across 24 h has a profound effect on behaviour and brain activity, and also influences the dynamics of waking and sleep. It has been previously shown that wheel availability substantially affects the amount and distribution of vigilance states, such that animals would fall asleep later and have less sleep altogether when they are given free access to the wheel^29^,^30^. On the contrary, if access to wheels is restricted to specific times of day, this can phase advance rhythms of behavioural activity, heart rate and body temperature, and can even improve these rhythms in mice with core circadian deficits^63^. Although the underlying mechanisms are unclear, the possibility of behavioural feedback affecting the circadian clock cannot be excluded, as suggested by studies where neuronal activity in the suprachiasmatic nucleus was recorded during voluntary RW activity^64^,^65^. Alternatively, our data suggest that stereotypic waking behaviours, such as wheel running may be associated with a ‘default' awake state, which in turn permits animals to stay awake longer and/or decrease sleep need. Arguably, this possibility would confer a significant advantage, as it would not only enable animals to remain effectively ‘online' and, therefore, avoid dangers associated with a sensory disconnection during sleep, but it may in some cases be necessary for specific behaviours such as during migration or a mating season when prolongation of wakefulness is favored ecologically^66^,^67^,^68^,^69^.

It has been suggested that sleep or sleep-like activity represents a default state of cortical networks, which not only provides benefits with respect to energy conservation, but is also essential for the maintenance of anatomical and functional connectivity among cortical and subcortical circuits^36^,^70^,^71^,^72^. Our data provide an important new angle to this notion, showing that waking behaviours consisting of repetitive stereotypic movement also share characteristics of a stable default state. We posit that an important feature of this state is that it may represent waking at a ‘lower cost', as manifested at the neuronal level by an attenuation of wake-dependent increases in cortical excitability. Such a low cost default wake state is uniquely suited to be effectively placed in the continuum between sleep and purposeful, goal-directed behaviours, and may render substantial adaptive flexibility with respect to changing environmental conditions, time of day and homeostatic needs.

## Methods

### Experimental animals

In total *n*=16 adult male mice, C57BL/6J strain, were used in this study (mean age 27.9±0.6 weeks at the time of the experiment). The animals were subdivided into two experimental groups, according to the cortical region where the microwire array was implanted (primary motor cortex, M1, *n*=11 and SCx, *n*=5). In several animals from the first group (*n*=5) more than one night has been analysed with the intention to ensure stability and reproducibility of results (1–3 days per animal). Subsequently, to prevent bias in selecting a specific day in the final analyses, for those animals where more than 1 day was analysed, the data were averaged between days before calculating means between animals. All mice were individually housed in custom-made clear plexiglass cages (20.3 × 32 × 35 cm) with free access to a RW (see below, Fig. 1, Supplementary Fig. 1). Cages were housed in ventilated, sound-attenuated Faraday chambers (Campden Instruments, Loughborough, UK, two cages per chamber) under a standard 12:12 h light–dark cycle (lights on 0900, ZT0, light levels ∼120–180 lux). Food and water were available *ad libitum.* Room temperature and relative humidity were maintained at 22±1 °C and 50±20%, respectively. Mice were habituated to both the cage and recording cables for a minimum of 4 days before recording. All procedures conformed to the Animal (Scientific Procedures) Act 1986 and were performed under a UK Home Office Project Licence in accordance with institutional guidelines.

### Surgical procedures and electrode configuration

Surgical procedures were carried out using aseptic techniques under isoflurane anaesthesia (3–5% induction, 1–2% maintenance). During surgery animals were head fixed using a stereotaxic frame (David Kopf Instruments, CA, USA) and liquid gel (Viscotears, Alcon Laboratories Ltd, UK) was applied to protect the eyes. One day before surgery animals received dexamethasone (0.2 mg kg^−1^, intraperitoneal) to suppress the local immunological response. Metacam (1–2 mg kg^−1^, subcutaneous (s.c.), Boehringer Ingelheim Ltd, UK) and dexamethasone (0.2 mg kg^−1^, s.c.) were administered preoperatively (and for at least 3 days after surgery). Post operatively animals were administered saline (0.1 ml per 20 g body weight, s.c.) and provided thermal support throughout and following surgery. A minimum 2-week recovery period was permitted before cabling the animals.

For this study, it was essential to maintain long-term stable recordings and unrestricted movement during spontaneous wheel running, so we avoided using large electrodes (for example, with a high channel count or laminar probes) or multiple arrays and probes. All mice were implanted with a single polyimide-insulated tungsten microwire array (Tucker-Davis Technologies Inc (TDT), Alachua, FL, USA). The arrays consisted of 16-channels (two rows each of eight wires) with properties as follows: wire diameter 33 μm, electrode spacing 250 μm, row separation L-R: 375 μm, tip angle 45 degrees. We used customized arrays where the lateral row of the wires was longer by 250 μm. A 1 × 2 mm craniotomy was made using a high-speed drill (carbon burr drill bits, 0.7 mm, InterFocus Ltd, Cambridge, UK) in the region of interest, with the midpoint of the craniotomy relative to bregma as follows: M1: anteroposterior +1.5–2 mm, mediolateral ∼2 mm; SCx: anteroposterior -1 mm, mediolateral 3.25 mm. The dura was dissected using a 25 gauge needle and saline was applied to the cranial opening to keep the exposed brain moist. In most cases, removal of the dura did not cause bleeding (when bleeding occurred, it was stopped with gelfoam soaked in sterile saline). The electrode array was advanced into the brain until the longer row of microwires was at the level of cortical layer 5 (M1: ∼0.7–0.8 mm below the pial surface, SCx: ∼0.5–0.6 mm). A two-component silicon gel (KwikSil; World Precision Instruments, FL, USA) was used to seal the craniotomy and protect the surface of the brain from dental acrylic. After 5–10 min, required for the gel to polymerise, dental acrylic was applied to fix the array to the skull. In all animals, EEG screws were placed in the frontal (motor area, anteroposterior +2 mm, mediolateral 2 mm) and occipital (visual area, V1, anteroposterior −3.5–4 mm, mediolateral 2.5 mm) cortical regions contralateral to the arrays using procedures previously described^73^. A reference and ground screw electrodes were placed above the cerebellum and an additional anchor screw was placed behind or opposite to the array to ensure implant stability. EEG screws were soldered (before implantation) to custom-made headmounts (Pinnacle Technology Inc. Lawrence, USA) and all screws and wires were secured to the skull using dental acrylic. Two single stranded, stainless steel wires were inserted either side of the nuchal muscle to record electromyography (EMG). A schematic diagram of implantation locations is shown in Supplementary Figs 1,3 and 5.

### Signal processing and analysis

Data acquisition was performed using the Multichannel Neurophysiology Recording System (TDT, Alachua FL, USA). Extracellular neuronal spike data were collected continuously (25 kHz, 300 Hz–5 kHz) concomitantly with LFPs from the same electrodes, and cortical EEG was recorded from frontal and occipital derivations. EEG/EMG data were filtered between 0.1–100 Hz, amplified (PZ5 NeuroDigitizer pre-amplifier, TDT Alachua FL, USA) and stored on a local computer at a sampling rate of 256.9 Hz. LFP/EEG/EMG data were resampled offline at a sampling rate of 256 Hz. Signal conversion was performed using custom-written Matlab (The MathWorks Inc, Natick, Massachusetts, USA) scripts and was then transformed into European Data Format using open source Neurotraces software (www.neurotraces.com). For each recording, EEG and LFP power spectra were computed by a Fast Fourier Transform routine for 4-s epochs (fast Fourier transform routine, Hanning window), with a 0.25 Hz resolution (SleepSign Kissei Comtec Co, Nagano, Japan).

### Scoring and analysis of vigilance states

While recordings were performed continuously, for the final analyses 1–3 undisturbed spontaneous 12-h dark periods were selected for each mouse, based upon the presence of several long consolidated waking periods dominated by RW-activity. Vigilance states were scored offline through manual visual inspection of consecutive 4-s epochs (SleepSign, Kissei Comtec Co, Nagano, Japan). Two EEG channels (frontal and occipital), EMG and RW activity were displayed simultaneously to aid vigilance state scoring. Vigilance states were classified as waking (low voltage, high frequency EEG with a high level or phasic EMG activity), NREM sleep (presence of EEG slow waves, a signal of a high amplitude and low frequency) or REM sleep (low voltage, high frequency EEG with a low level of EMG activity). Great care was taken to eliminate epochs contaminated by eating, drinking or gross movements resulting in artifacts in at least one of the two EEG derivations or in any of the MUA channels (11.5±3.2 % of total recording time). Detailed analyses of neuronal activity (see below) were based on selected time intervals to ensure stability of neuronal waveforms within the time window specified. The total period included in the analyses was on average 101.1±7.7 min, and consisted of 96.3±7.4 min spent awake (of which 41.0±3.7% of time was spent running). For the analyses on wake-dependent changes in firing rates, consolidated waking periods occurring throughout the experimental night were used. These were defined as continuous waking periods lasting at least 40 min that were not interrupted by periods of sleep >3 min. On average 4.6±0.5 such waking periods were detected per animal per 12-h, which lasted on average 118.0±14.2 min (95 in total in *n*=16 mice, Supplementary Fig. 11). Within each of these wake periods, the first and the last 15 min intervals, referred to as W1 and W2 (Fig. 5), were analysed. These consisted of 14.7±0.2 and 14.6±0.2 min of waking respectively. To exclude the effects of running of neuronal activity during these intervals, these analyses included nwRUN waking only, which occupied most of W1 and W2 intervals (wRUN waking during W1 and W2 averaged 2.0±0.3 min only, and the amount of running was not significantly different between W1 and W2. Since neuronal activity immediately after awakening is known to be decreased as compared with average firing rates during the waking episode^74^, we also performed this analyses after excluding the first and the last 3 min of each waking period, which did not noticeably affect the results.

### Sleep deprivation

In a subset of animals, in which the microwire array was implanted in M1 (*n*=9), sleep deprivation was performed for 6 h starting at light onset. Sleep deprivation was performed in order to investigate whether the relationship between wheel running and cortical activity is affected by lighting conditions, the time of day, preceding sleep-wake history and exposure to novel objects. During sleep deprivation, the animals were awake 98.9±0.3% of time, and their behaviour and polysomnographic recordings were under constant visual observation. Sleep deprivation was performed in the animal's home cage, where they had free access to RWs throughout the procedure, which they used intermittently (Supplementary Fig. 9). Throughout the sleep deprivation procedure, the animals were regularly provided with novel objects to mimic naturalistic conditions of wakefulness an in ethologically relevant manner^75^. All mice were well habituated to the experimenter and to the exposure to the novel objects before the experiment. Novel objects included nesting and bedding material from other cages, wooden blocks, small rubber balls, plastic, metallic, wooden or paper boxes and tubes of different shape and colour.

### Verification of recording sites

In this study, we performed LFP and MUA recordings using microwire arrays, which were aimed for deep layers of the primary motor cortex (*n*=11) and somatosensory area (*n*=5). To confirm the location of the arrays, upon completion of the experiments mice were deeply anaesthetized and transcardially perfused with 0.9% saline followed by 4% paraformaldehyde solution. Brains were photographed, and the location of the exact site of implantation was calculated. The brains were subsequently sectioned using a freezing Microtome (Leica, Germany) to produce 50 μm coronal slices throughout the region of interest. Sections were mounted on slides and subjected to cresyl-violet (Nissl) staining. For a subset of animals, microwire arrays were coated with a thin layer of DiI fluorescent dye (DiIC18(3), Invitrogen) to facilitate subsequent localization^76^. In this study, we did not intend to investigate the laminar profile of neuronal activity. Instead, we aimed at recording spiking activity from a sufficiently large area of the neocortex, spanning 1.75 mm in the antero-posterior dimension and 375 and 250 μm in the lateral-medial and dorsal-ventral dimensions, respectively. In M1 this allowed us to span various parts of the motor cortex representing different body parts, such as the hindlimb, forelimb and trunk^31^. Within the SCx, subsequent analysis revealed that > 80% of all wires were located within the barrel cortex, and >90% of wires within the broader SCx. In one individual mouse most posterior wires were presumably located at the border of V2. However, because of well-known anatomical variability between individual animals, and since it was not possible to perform mapping of cortical regions in our study, the exact location of the arrays with respect to precise borders or functionally differentiated cortical areas cannot be ascertained. In this study, we observed a generalized decrease of cortical spiking activity during high speed stereotypic running. To investigate the influence of electrode location on the effects we observed, we performed detailed comparison between the longer and shorter row of wires within the array, and between wires along the antero-posterior direction. Concerning the comparison between longer and shorter wires of each array, the analysis did not reveal any noticeable differences with respect to slow-wave triggered average or running-speed dependency of neuronal spiking (Supplementary Fig. 12). This result is consistent with our histological analysis (representative sections are shown in Supplementary Figs 3 and 5), which indicated that in either region both rows of wires were located predominantly within layer 5, although careful inspection revealed that in some cases the shorter wires terminated on the border between layer 4 and 5, while the longer wire reached the border between layer 5 and layer 6a. To investigate whether the changes in neuronal activity are influenced by the position of individual recording wires within the array along the anterior-posterior direction, we grouped the 16 wire electrodes into four clusters (each of four wires) for both regions (Supplementary Fig. 4), and calculated the average firing rates, as well as the proportion of RUN off neurons (neurons which decrease their spiking during running) independently for each of the four clusters. This analysis revealed that in most recording locations neuronal activity decreased during wRUN waking, as compared to nwRUN waking, although, especially in SCx, this effect was more pronounced in more anterior locations.

### LFP waves in wake and NREM sleep

The relationship between neuronal activity and LFP signals (Fig. 4, Supplementary Figs 10 and 12) was analysed in a subset of animals (M1, *n*=7; SCx: *n*=5) as previously^55^. The LFP signal was band pass filtered (wake: 2–6 Hz, stopband edge frequencies 1–8 Hz; NREM sleep: 0.5–4 Hz, stopband edge frequencies 0.1–8 Hz) with MATLAB filtfilt function exploiting a Chebyshev Type II filter design (MATLAB, The Math Works, Inc., Natick, MA)^20^,^77^, and waves were detected as positive deflections of the filtered LFP signal between two consecutive negative deflections below the zero-crossing. Only LFP waves with the peak amplitude larger than the median amplitude across all detected waves within the same vigilance state were included. Calculating average MUA triggered by the LFP waves revealed that positive LFP waves during NREM sleep were associated with a reduction of MUA (Fig. 1c, Supplementary Figs 10 and 12). In contrast, during waking, in most animals, in which recordings were performed from M1, MUA did not show, on average, a systematic relationship with LFP waves, although a modest reduction in MUA during positive LFP waves was typically observed in SCx, which occurred within an ∼200 ms window around the positive peak of the wave. Visual inspection of the raw traces during waking, revealed an occurrence of neuronal silence accompanied with positive LFP waves (Suppl. Movie 5), which in most cases encompassed a subset or one recording channel only, which likely explains the lack of overall relationship between overall MUA and LFP in active animals.

### Running-wheel activity

RW activity was the main behaviour investigated in this study. It is a widely used behavioural assay, utilizing the well-established fact that mice have a spontaneous tendency to run. We used standard, rather than complex wheels, since the main aim of this study was to investigate the effects of highly stereotypic automatic running, rather than motor learning. In this study, animals had free access to RWs (Campden Instruments, Loughborough, UK, wheel diameter 14 cm, bars spaced 1.11 cm apart inclusive of bars) for 15.3±2.1 days before the analysed dark periods, and were, therefore, well adapted to the wheels. The wheels were custom made for tethered animals, and did not prevent the animals from running ad libitum. There was no significant difference in the duration of the habituation to the wheel between the groups of animals (unpaired *t*-test: M1 versus SCx, *P*=0.21). Each RW was fitted with a digital counter (Campden Instruments), which uses an infra-red emitter/receiver to detect each rung passing the infra-red beam as the wheel rotates. In our study RW activity was recorded with a high temporal resolution (one full wheel revolution consists of 38 individually detected rung counts, thus 10 counts per second corresponds to 10.11 cm s^−1^) within the same system used to record electrophysiological signals. This allowed us to achieve a precise synchrony between instantaneous changes in electrophysiological signals/behaviour and RW activity/speed. The wheel counter output is a 5V TTL pulse (0V with no output), that triggers an edge detector in the TDT acquisition system, and in turn creates a time stamp that is stored for each wheel count. For most analyses, RW-activity was analysed in 1-s epochs that were grouped based on the number of counts during the corresponding epochs. The average running speed for those epochs with at least 1 count was 9.6±0.6 counts per second. Since the number of 1-s epochs with progressively higher numbers of counts decreased progressively, we also used progressively wider ranges for the bins (0; 1; 2–3; 4–7; 8–11; 12–32), to obtain a sufficiently high yet reliable number of counts within each speed category (Supplementary Fig. 2d). Since the number of 1-s epochs for non-running waking significantly exceeded the number of epochs with RW-activity, some of the analyses were repeated on a randomly chosen subset of non-running epochs matching the number of epochs with RW activity, which did not noticeably affect the results.

Two animals in the motor cortex implanted group were excluded from specific data analyses where the effects of RW speed were calculated, due to a low temporal resolution of RW-activity recordings (one for technical reasons and the other because an individual mouse never attained high speed running). Therefore, only *n*=9 mice contributed to these mean values. For the analyses depicted on Fig. 3, we quantified neuronal firing rates during epochs where the animals accelerated on a wheel, ran steadily or decelerated. This analysis has been performed on a 1-s basis, focusing on those epochs with at least four RW counts. To obtain an ADI we calculated mean second derivative of the time series corresponding to the timings of consecutive counts for each 1-s epoch, and subsequently grouped epochs according to ADI from largest negative (acceleration) to largest positive (deceleration) values into ten 10% deciles before calculating averages between animals (Supplementary Fig. 7). As a result, the first 10% decile corresponds to fastest acceleration, the tenth decile—to fastest deceleration, while the middle of the distribution (deciles 5–6) correspond mostly to steady running with a close to zero net speed change (Fig. 3, Supplementary Fig. 7).

### Analysis of extracellular neuronal activity

This study was triggered by a finding of a conspicuous decrease in MUA recorded from the primary motor cortex, which we noticed based on visual observations of raw signals (Supplementary Movies 1–4). Our analyses are based on extracellular recordings of MUA, which on one hand allows recordings of activity from a relatively large neuronal population and on the other hand enables long-term relatively stable recordings in freely moving animals. Online spike sorting was performed primarily to eliminate artifactual waveforms caused by electrical or mechanical noise. This was performed with OpenEx software (TDT), by manually applying an amplitude threshold for online spike detection. Whenever the recorded voltage exceeded this predefined threshold (2 × > noise level, at least −25 μV), a segment of 46 samples (0.48 ms before, 1.36 ms after the threshold crossing) was extracted and stored for later use, together with the corresponding time stamps. On average, 9.5±0.8 channels per animal showed robust MUA activity and were included in the final analyses. Although we observed that overall neuronal firing rates on average decreased with increasing running speed, we observed putative single units, which did not show visually detectable changes or even showed an opposite trend (for example, Supplementary Figs 3 and 5; Supplementary Movies 1–4). Therefore, to investigate the relationship between running behaviour and individual putative single units' firing, we employed offline spike sorting. Firstly, an artifacts removal procedure was implemented in order to eliminate remaining artifactual waveforms and to facilitate subsequent clustering. Typical spurious spike waveforms included waveforms, which were likely the result of a combination of several spikes from different neurons occurring concurrently and preventing their reliable classification. After removal of the artifactual spikes, principal components were computed using principal component analysis (PCA) on a segment of the spike waveform between the 5th and the 35th time sample, because this is the segment that contains the initial negative peak after threshold crossing and subsequent after hyperpolarization, and is, therefore, more informative about the overall spike waveform. In PCA an orthogonal linear transformation of the original data is performed, through singular value decomposition of the data matrix^78^. After performing PCA, each spike between samples 5 and 35 was thus described by 31 variables, each being a linear combination of the original sampling values. Each principal component (PC) corresponds to an eigenvalue and an eigenvector from the singular value decomposition of the data matrix. The first PC is associated with the eigenvector on the direction of maximal variance of the data set, and so on in decreasing order for the remaining PCs. The ratio between each eigenvalue and the sum of all eigenvalues represents the ratio of the total variance described by a single PC.

Clustering was performed based on the k-means algorithm^79^. This is a partitioning method that aims to divide *n* observations into *k* clusters in which each observation belongs to the cluster with the nearest mean, serving as a prototype of the cluster. There are several k-means algorithms available, which are based on different ways to proceed through the iterations. We used the k-means function in Matlab, which was implemented according to Lloyd's algorithm^80^. In particular, it chooses *k* initial cluster centers (centroid) through an initialization step, and then computes point-to-cluster-centroid Euclidean distances of all observations to each centroid. Based on the distance, each observation is assigned to the cluster with the closest centroid. As a next step, the algorithm computes the average of the observations in each cluster to obtain *k* new centroid locations. Finally, it repeats previous steps until cluster assignments do not change, or the maximum number of iterations is reached. This approach requires the user to a priori select the number of clusters. Based on visual inspection, it was found unlikely that more than five distinct waveforms were present in the same MUA channel, and so the procedure has been performed with *k*=2, 3, 4 and 5. For each case, several features were extracted within each cluster and were used to validate the quality of clustering and to select the best outcome of the clustering procedure within each animal and recording channel. These were the average spike waveform with standard deviation, interspike interval distribution, the time course of peak-to-peak amplitude across the recording period, the corresponding time course of average firing rates and the autocorellogram of the spike trains. As a result, on average 3.2±0.1 clusters per recording channel were obtained. All selected clusters were screened and visually classified according to their signal to noise ratio, waveshape of the action potential, stability of the amplitude across time and ISI distribution histogram, and spurious or unstable clusters were excluded from the analysis. As a result, 1.9±0.1 and 2.5±0.1 putative single units per recording channels were retained for the final analyses in M1 and SCx implanted animals, respectively. We should emphasize that based on our recording and sorting technique it cannot be excluded that two or more neurons having identical spike waveform shapes, amplitude and firing properties are present simultaneously on the same channel. However, we often observed that individual neurons classified as ‘putative single units' changed their firing during running behaviour in a highly consistent manner within the recording session or even across continuous days (Supplementary Fig. 13). We claim that the presence of such functional tuning to behaviour is important evidence that at least in the majority of cases our spike sorting procedure was successful at yielding single units. Since putative single units varied greatly in absolute firing rates from <1 Hz to more than 60 Hz (Supplementary Fig. 14), we avoided, in most cases, to average absolute values. We argue that in this case mean values would be biased by high-frequency spiking putative single units, even if slow-spiking neurons show relatively more pronounced changes. One way to account for this is to express firing rates of putative single units as a percentage of their mean firing rates across all artifact free epochs within the time interval of interest, as we have done for most analyses.

### Statistical analysis

All statistical analyses were performed in Matlab (The MathWorks Inc, Natick, Massachusetts, USA) and all values reported are mean±s.e.m. ANOVAs for repeated measures were used to detect statistically significant effects of RW speed or acceleration on firing rates. Two-tailed paired *t*-tests were used for comparisons. In addition, we also employed a normalization procedure by expressing some of the variables in relative terms (as % of the mean value across all artifact free epochs during the recording period). We have done this in those cases where interindividual variability of absolute values is not relevant for the effects observed, as it allowed us to assess the relative magnitude of the effects. Normalization of values in this manner is a widely used approach and, therefore, facilitates comparison between studies, where relative values are reported.

### Data availability

All relevant data will be available from the authors upon request.

## Additional information

**How to cite this article**: Fisher, S. P. *et al*. Stereotypic wheel running decreases cortical activity in mice. *Nat. Commun.* **7**, 13138 doi: 10.1038/ncomms13138 (2016).

## Supplementary Material

Supplementary Information Supplementary Figures 1 - 14

Supplementary Movie 1 Modulation of cortical MUA by voluntary wheel running. Raw spike recording of four channels of multi-unit activity (MUA) in the motor cortex of one individual freely behaving, non-head-fixed mouse. The screen width corresponds to a 20-second record. Bottom bar plot corresponds to running wheel activity (each vertical bar represents a single count of the wheel rung). Note that initially before running the MUA activity is relatively high and variable in all channels. There is a substantial slowing down or cessation of spiking activity particularly in channels 2-4 upon the initiation of intense running. Yellow numbered tick marks at the top represent 1 second segments. Bottom bar plot corresponds to running wheel activity (each vertical bar represents a single count of the wheel rung).

Supplementary Movie 2 RUN-ON and RUN-OFF neurons during voluntary wheel running. Raw spike data displaying three channels of MUA recorded from the same 16-ch microwire array in the motor cortex in an individual mouse. This example illustrates the differential modulation of individual neurons in the motor cortex by voluntary running wheel behaviour in a non-head-fixed mouse. The green MUA channel (2nd trace from the top) initially exhibits irregular fast spiking activity during a non-running waking period which is inhibited upon the onset of running ("RUN-OFF" neuron). In contrast, the blue MUA raw trace (3rd trace from the top) exhibits a brief surge of intense spiking at running onset followed by continuous irregular activity throughout the running bout ("RUN-ON" Neuron). Yellow numbered tick marks at the top represent 1 second segments. Bottom bar plot corresponds to running wheel activity (each vertical bar represents a single count of the wheel rung).

Supplementary Movie 3 Stereotypic running suppresses MUA. Raw spike data from four MUA channels recorded in the motor cortex of one individual mouse. This example highlights the effects of the pattern of running wheel activity on cortical MUA. Bottom bar plot corresponds to running wheel activity (each vertical bar represents a single count of the wheel rung). Note that neurons in the top two traces (shown in blue and red) are mostly inactive during high speed steady running (as indicated by a high density of vertical lines on the bottom bar plot), but increase activity when the running pattern deviates from a stereotypic form, such as during brief periods of slowing down or a cessation of running. Yellow numbered tick marks at the top represent 1 second segments.

Supplementary Movie 4 Firing activity associated with brief spontaneous interruptions in voluntary wheel running. Raw spike data depicting two traces of MUA recorded in the motor cortex during an episode of running wheel activity. Bottom bar plot corresponds to running wheel activity (each vertical bar represents a single count of the wheel rung). Note the correlation between the pattern of running and the activity of the neuron depicted on the top channel (shown in yellow). Specifically, MUA exhibits transient periods of high frequency firing concomitant with brief pauses in running wheel activity, and these surges in firing are largely absent during high speed running. Yellow numbered tick marks at the top represent 1 second segments.

Supplementary Movie 5 LFP and MUA in an awake mouse during voluntary wheel running. Top: Raw electroencephalogram (EEG) signals recorded from the frontal (yellow) and occipital (red) cortex together with neck muscle electromyogram (EMG). Middle: Raw traces of local field potentials (LFP) and corresponding multiunit activity (MUA) in the same seven channels recorded from the somatosensory cortex in a representative freely behaving mouse during a period of running wheel activity. Bottom bar plot corresponds to running wheel activity (each vertical bar represents a single count of the wheel rung). Note the occurrence of brief isolated periods of reduced MUA that coincide with positive LFP waves during high speed running. The first six examples are highlighted with arrows. The screen width corresponds to a 5-second period, and yellow numbered tick marks at the top represent 1 second segments.
