# Supplementary material for: Stereotypic wheel running decreases cortical activity in mice
Source: Nat Commun. 2016 Oct 17;7:13138. doi: 10.1038/ncomms13138 (PMC5071642; doi:10.1038/ncomms13138)
Supplement: Supplementary Information — Supplementary Figures 1 - 14 [file ncomms13138-s1.pdf]

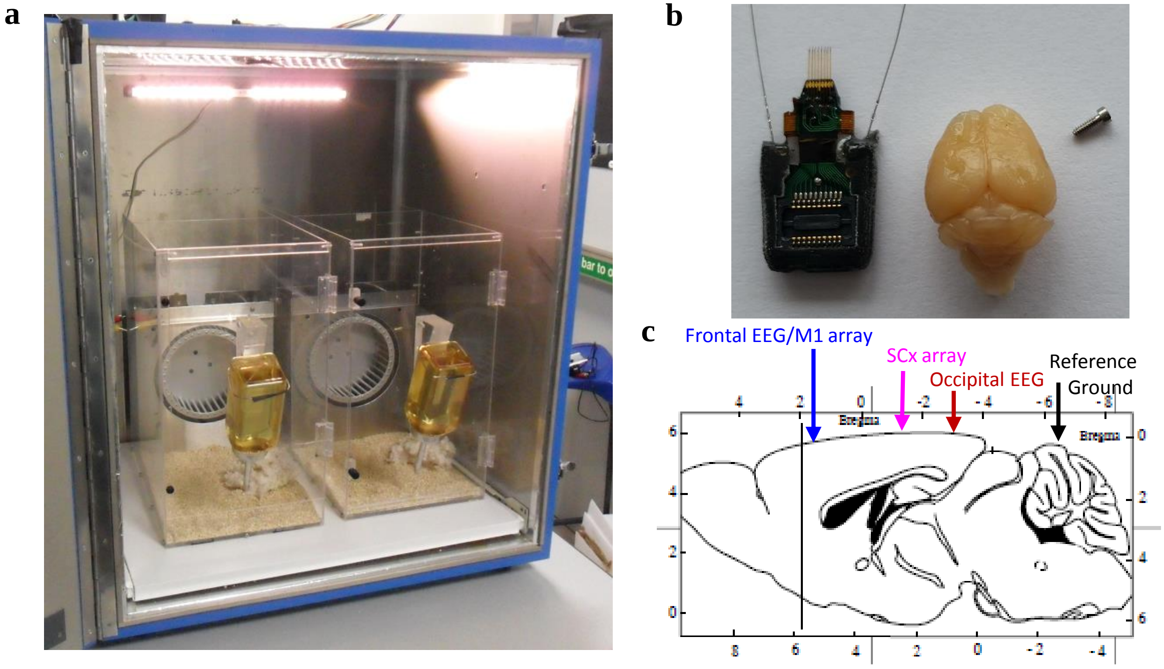

Supplementary Figure 1. Experimental set up and electrode positions. (a) Animals were individually housed in custom-made home cages, providing continuous free access to a running wheel, inside ventilated, sound-attenuated chambers provided with autonomous light-dark (12:12) control. **(b)** A photograph of a sample 16-ch microwire array, which were used in this study for local field potentials (LFP) and multiunit activity (MUA) recordings in all animals, and a representative stainless screw used to record EEG signals and as a reference and ground. Both devices are shown alongside the mouse brain to illustrate relative size proportions. **(c)** Schematic depiction of the positions of the 16-ch microwire arrays used to record MUA and LFP in the primary motor cortex (M1) and somatosensory cortex (SCx), the frontal and the occipital EEG screws and the position of reference and ground screws placed above the cerebellum.

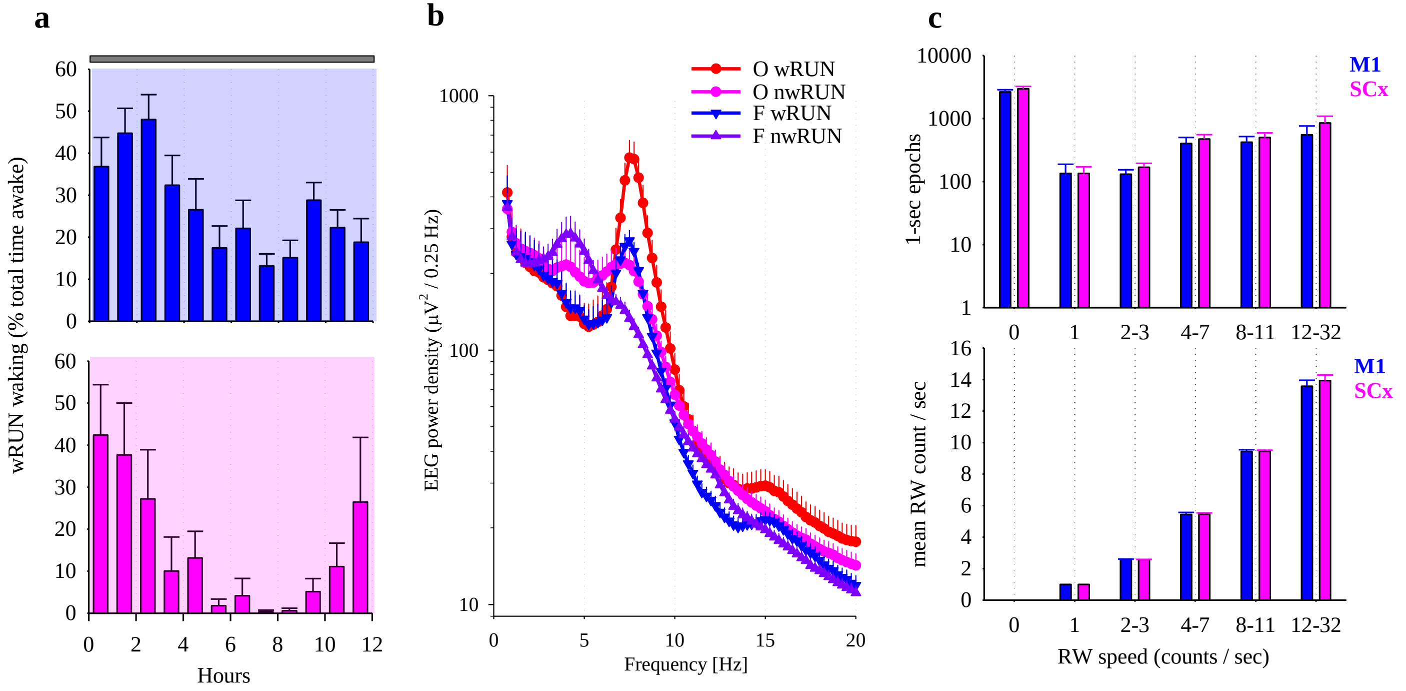

Supplementary Figure 2. (a) Average profile of running-wheel (RW) activity during the experimental night. From top to bottom: the amount of waking spent running in animals implanted in the motor cortex (M1, n=11, blue) and somatosensory cortex (SCx, n=5, pink). Mean values, s.e.m. **(b)** EEG power spectral density during running (wRUN) and non-running (nwRUN) waking shown for the frontal (F) and occipital (O) EEG signals. Mean values, s.e.m. (n=11 mice). **(c)** Top: The total number of 1-sec epochs with and without running which contributed to this study shown for the experiments where cortical firing rates were investigated in M1 and SCx. All epochs were grouped as a function of increasing running speed. Bottom: The corresponding average values of RW speed (counts / sec). Mean values, s.e.m., n=11 mice (M1) and n=5 mice (SCx).

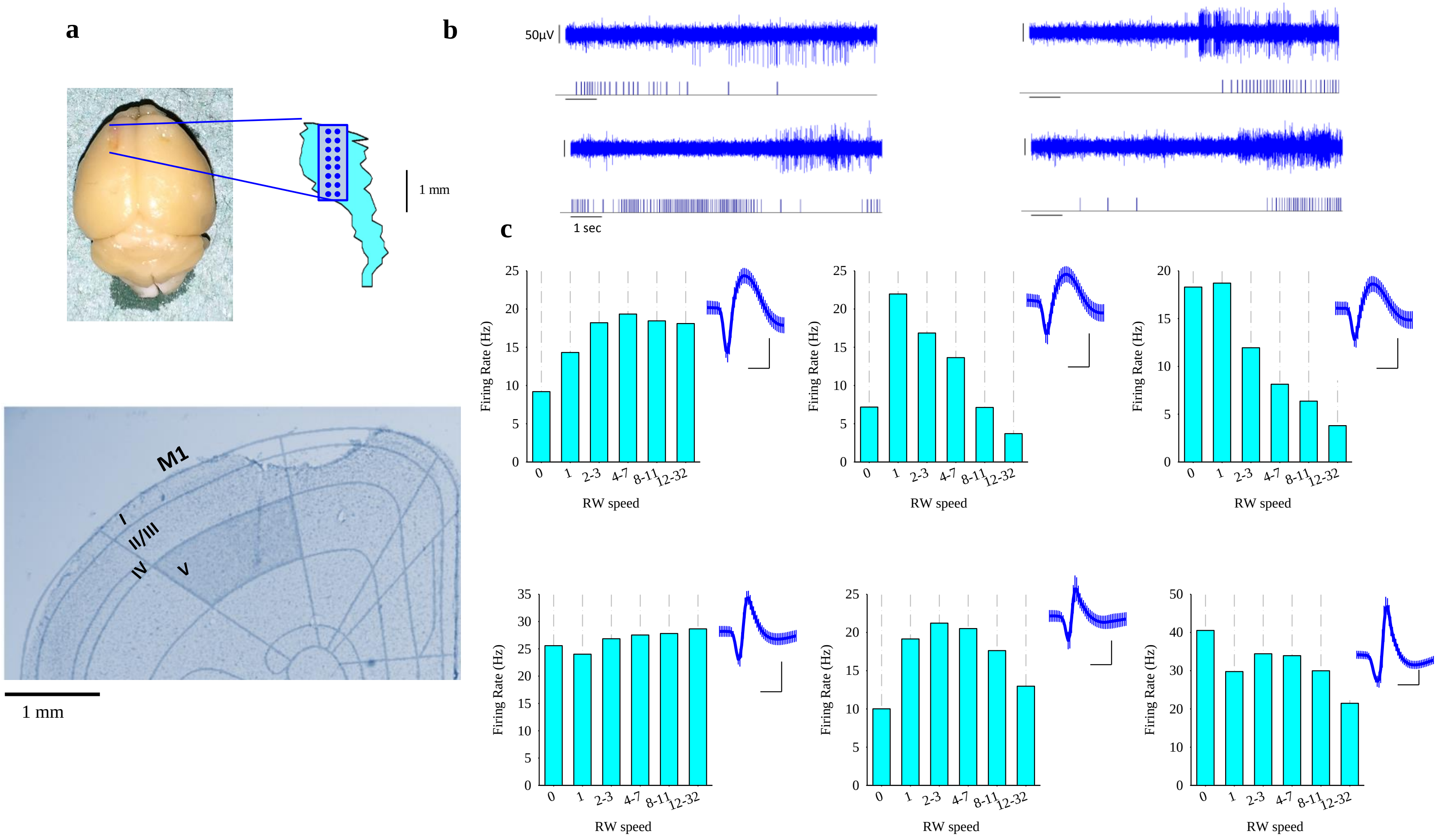

Supplementary Figure 3. **(a)** Right: A photograph of a representative brain taken from a mouse, in which the 16-ch microwire array was placed in the left primary motor cortex (M1). Left: The location of a 16-ch microwire array (two rows, each of 8 wires) is schematically depicted within M1 based on coordinates taken from Paxinos & Franklin, 2001. Bottom: Representative histological verification (Nissl staining) of the electrode location in M1 (one single wire tract is shown with an overlay of an anatomical reference adapted from the Allen Mouse Brain Atlas). **(b)** Individual representative examples depicting the effect of voluntary running on cortical neuronal activity in M1. Four neurons are shown recorded from M1 together with corresponding running wheel (RW)-activity (each bar represents a single wheel rung count). The two examples on the left depict cases when multiunit activity (MUA) decreases specifically during running. The two examples on the right show traces, where MUA increases immediately before running onset and is maintained during running. **(c)** Individual examples of single putative neurons recorded in M1 (six in total, taken from different animals) showing the average spike waveform (±standard deviation) and the change in average firing rates as a function of running speed. Scale bars: vertical: 50µV, horizontal: 0.5 ms. RW speed refers to the number of counts / sec as shown on Supplementary Fig. 2d (x-axis). Note that individual neurons in M1 were variable with respect to their firing as a function of running speed.

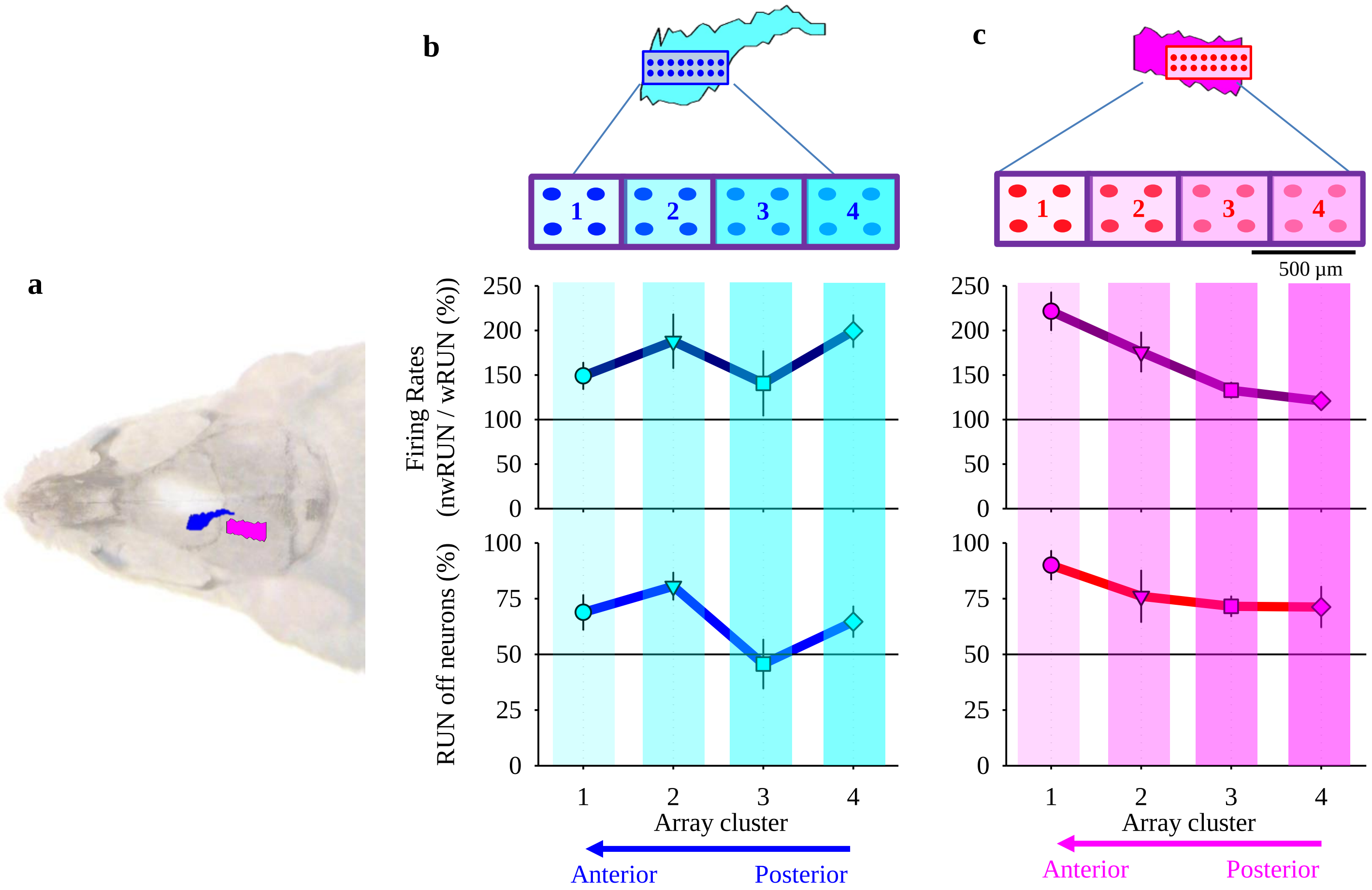

Supplementary Figure 4. Anterior-posterior gradient in the change of cortical neuronal activity as a function of voluntary wheel running. **(a)** The position of the primary motor cortex (M1, blue) and somatosensory cortex (SCx, pink) shown on the dorsal surface of the mouse head. **(b)** Top: Schematic depiction of the electrode positions on the 16-channel array colour coded along the anterior-posterior axis. All 16 wires within the array were subdivided into four clusters (1-4). Bottom: The relationship between the wire electrode position on the array and neuronal activity as a function of running behaviour, represented as relative firing rates during nwRUN waking as % of wRUN waking, and below the proportion of RUN off neurons as % of all recorded neurons. Mean values, s.e.m., n=9 mice. **(c)** The same for SCx. Mean values, s.e.m., n=5 mice.

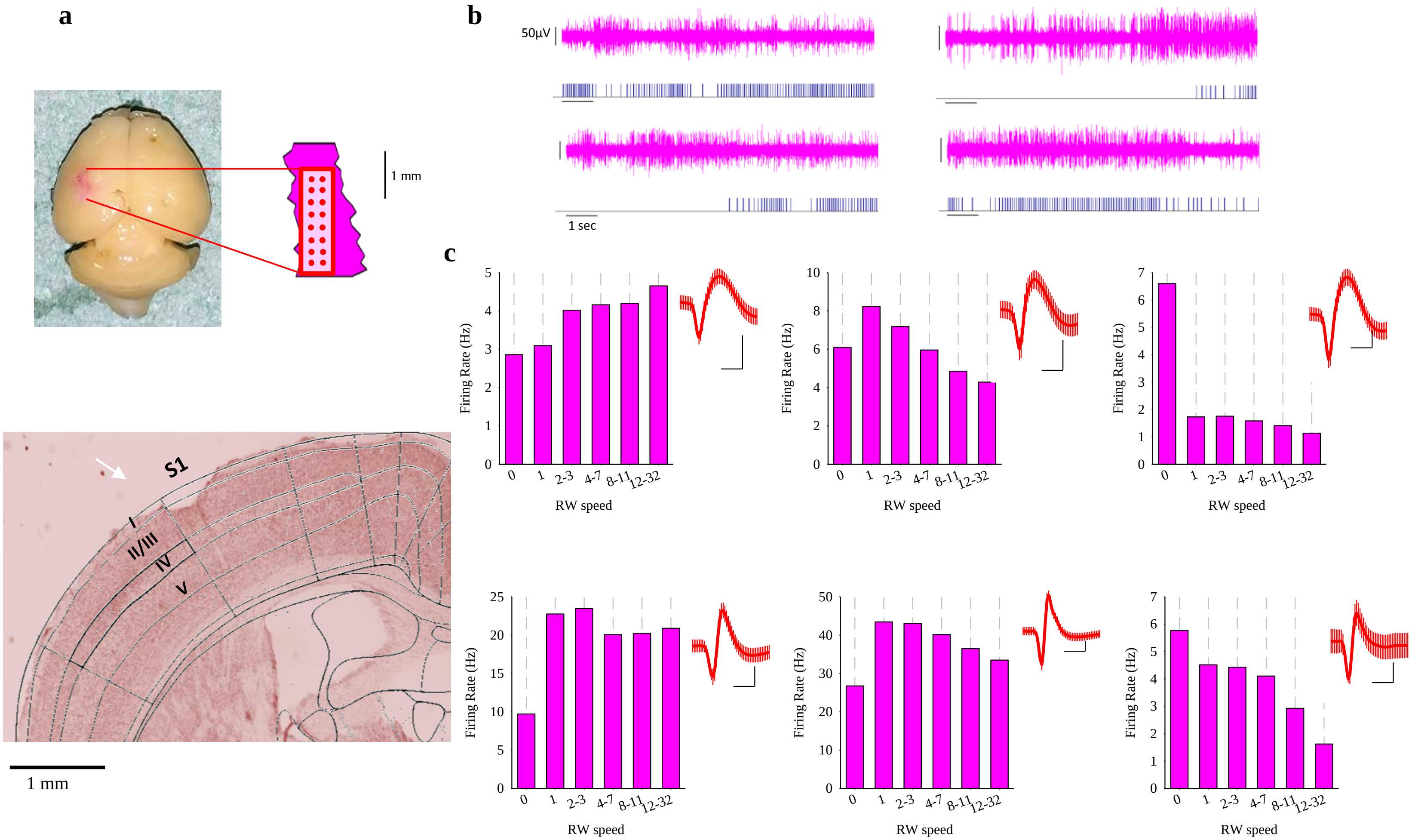

Supplementary Figure 5. (a) Left: A photograph of a representative brain taken from a mouse, in which the 16-ch microwire array was placed in the left somatosensory cortex (SCx). Right: The location of a 16-ch microwire array (two rows of 8 wires each) is schematically depicted within the barrel cortex based on coordinates taken from Paxinos & Franklin, 2001. Bottom: Representative histological verification (Nissl staining) of the electrode location in SCx (one single wire is shown with an overlay of an anatomical reference adapted from the Allen Mouse Brain Atlas). **(b)** Individual representative examples depicting the effect of voluntary wheel running on cortical neuronal activity in SCx (four MUA traces); corresponding running wheel (RW)-activity is shown below. The two examples on the left depict cases when spiking activity decreases specifically during running. The two examples on the right show traces where multiunit activity (MUA) increases immediately before running onset and is maintained during running. **(c)** Individual examples of putative single neurons (six in total, taken from different animals) showing the average spike waveform (±standard deviation) and the change in their average firing rates as a function of running wheel speed. Scale bars: vertical: 50µV, horizontal: 0.5 ms. RW speed refers to the number of counts / sec as shown on Supplementary Fig. 2d (x-axis). Note that individual neurons in SCx were variable with respect to their firing rates as a function of running speed.

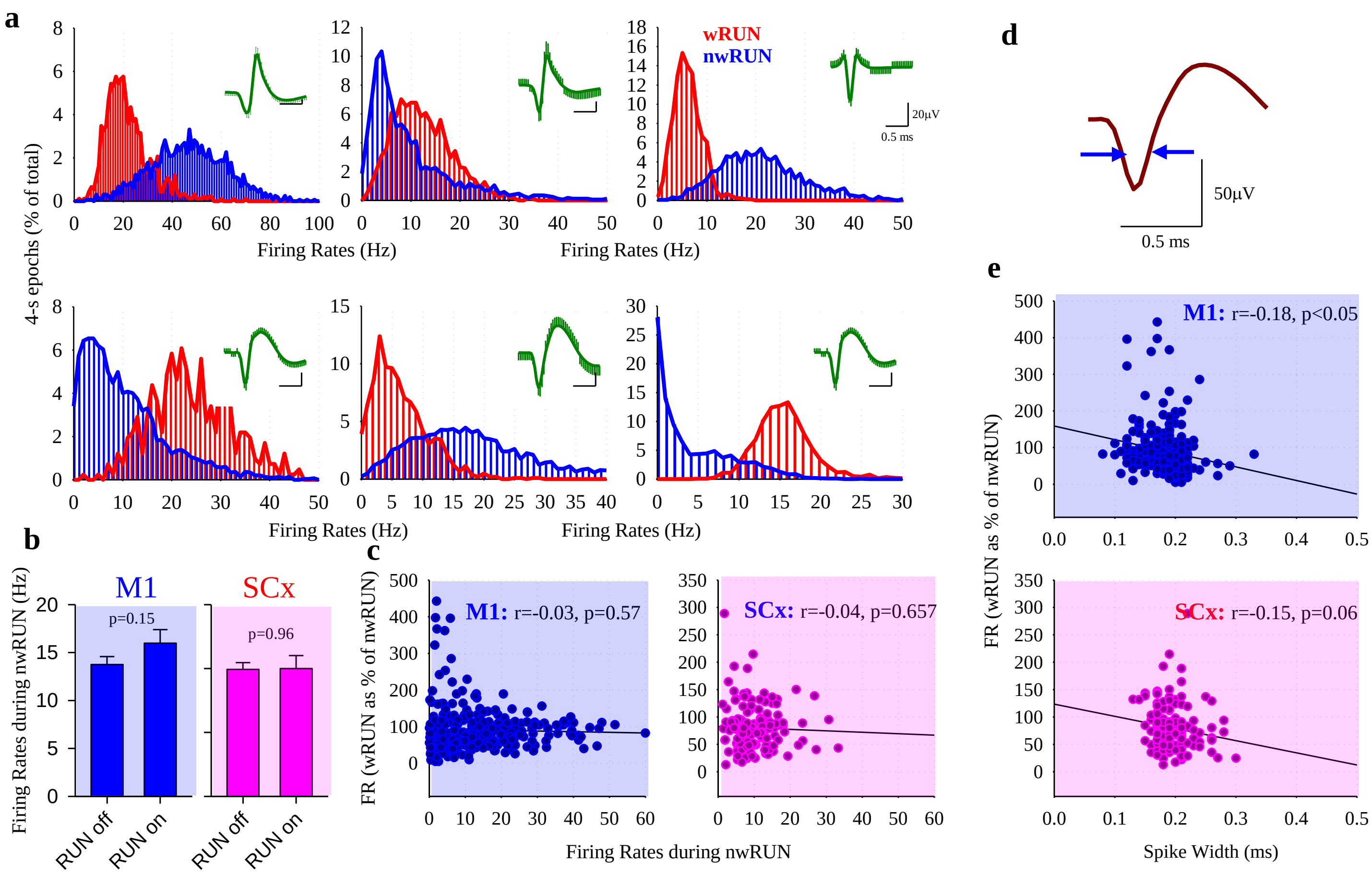

Supplementary Figure 6. (a) Distribution of 4-sec epochs as a function of firing rates during wRUN and nwRUN waking. Six individual putative single units are shown. Insets: corresponding spike wave shapes. Note that running is associated with a substantial shift in the distribution of firing rates. **(b)** Average firing rates during nwRUN waking calculated separately for putative single units, which decrease firing during running (RUN off) and those, which increase spiking during wRUN waking (as shown on Fig. 1e and 2c, mean values, s.e.m,, M1: n=9, SCx, n=5). **(c)** The relationship between average absolute firing rates during nwRUN waking and the change in firing rates during wRUN waking (as % of corresponding activity in nwRUN waking). Each dot represents an individual putative single unit. Straight line depicts linear regression. **(d)** Representative spike wave form illustrating the approach to calculate spike width. **(e)** The relationship between spike width and the change of firing rates from nwRUN to wRUN waking. Each dot represents an individual putative single unit. Straight line depicts linear regression.

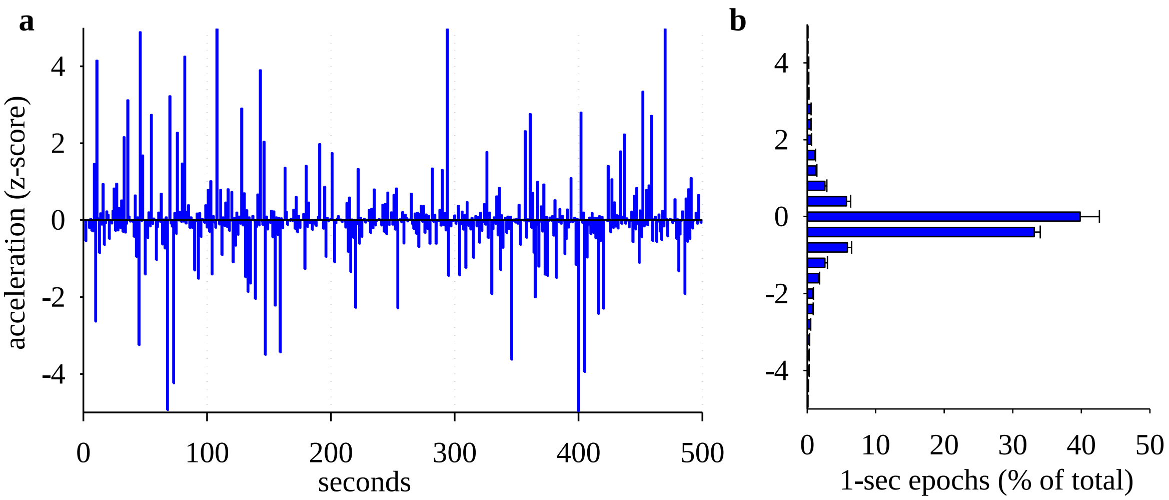

Supplementary Figure 7. The pattern of wheel running. (a) The distribution of 1-sec epochs with wheel acceleration (negative values) or deceleration (positive values) within a representative bout of running in one individual animal. **(b)** Distribution of 1-sec epochs as a function of wheel acceleration and deceleration (z-score). Note that during the majority of epochs, running is stereotypic as manifested in a relatively constant speed, although in a subset of epochs rapid changes in running speed are apparent. Mean values, s.e.m, n=9 mice.

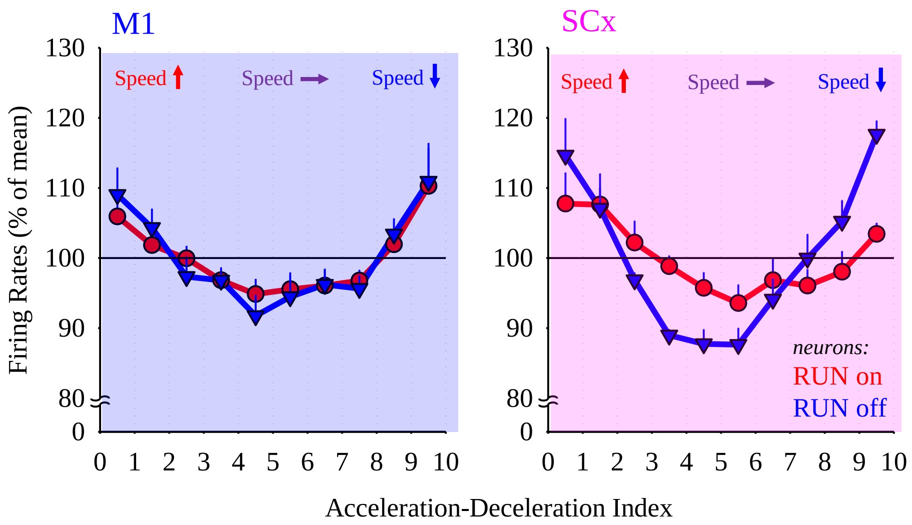

Supplementary Figure 8. The relationship between spontaneous acceleration and deceleration while running on the wheel and cortical firing rates. All 1-sec epochs were subdivided into ten 10% deciles, each consisting of the same number of epochs. These were sorted as a function of the change in running speed within an epoch from fast acceleration to fast deceleration (schematically shown as arrows above), and corresponding population firing rates were calculated prior to averaging between animals (M1: n=9 mice, SCx: n=5 mice, mean values, s.e.m.). Corresponding statistics are as follows: M1: RUN on, $F(9,89)=3.18$, 0.0027; RUN off, $F(9,89)=4.1$, 0.0003; SCx: RUN on, $F(9,49)=2.49$, $p=0.03$, RUN off, $F(9,49)=13.0$, $p=6.3261e-009$.

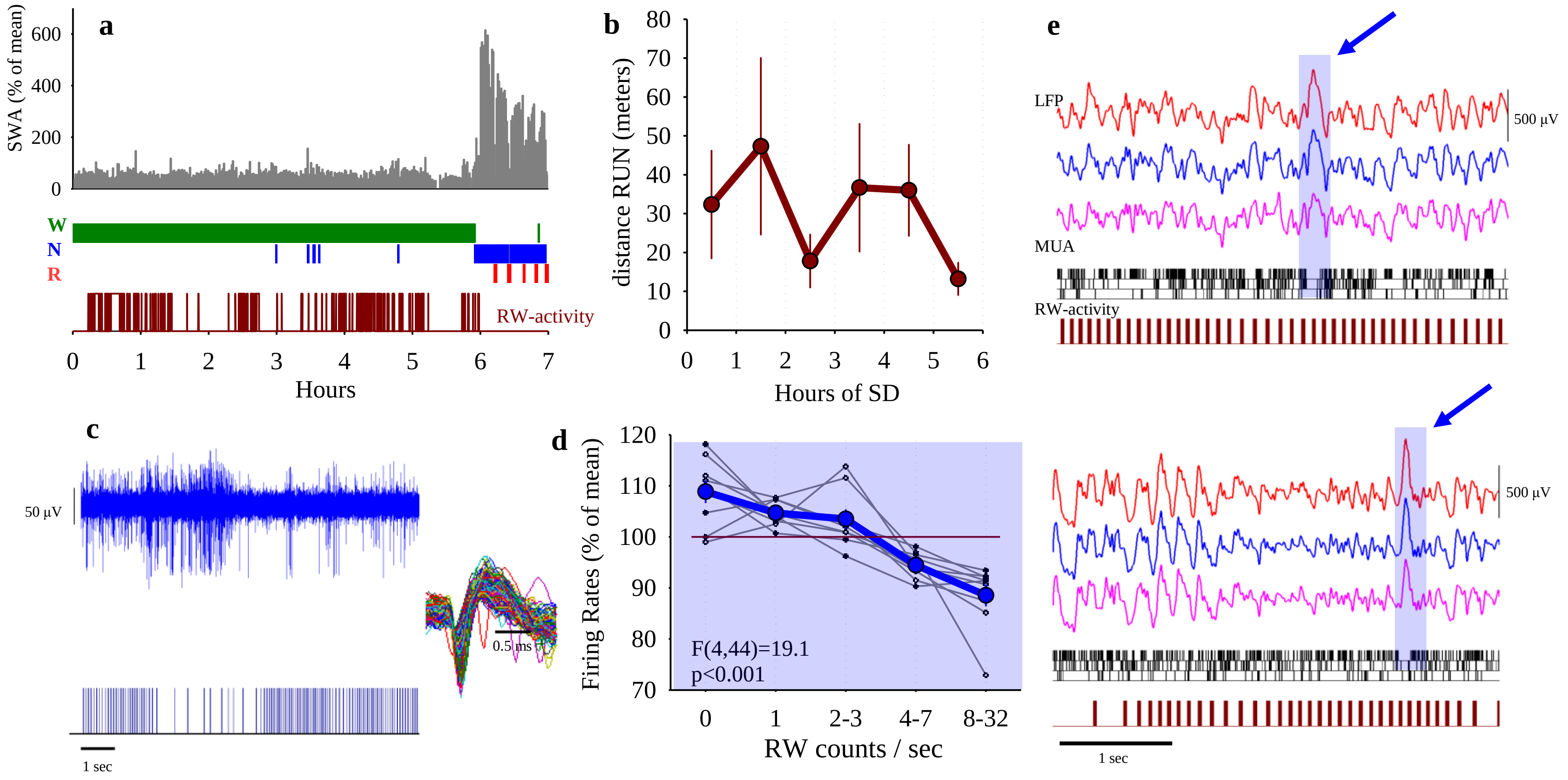

Supplementary Figure 9. Cortical neuronal activity in M1 during wheel running during sleep deprivation. (a) Individual profile of EEG slow-wave activity (SWA, EEG power between 0.5-4.0 Hz) recorded in the frontal cortex, running-wheel (RW) activity and the distribution of sleep-wake stages (W= wakefulness, N = NREM sleep, R= REM sleep) across 6-h of sleep deprivation (SD) and subsequent 1 hour of sleep in one representative mouse. **(b)** Time course of RW activity represented as distance run per hour during 6-h SD. Mean values, s.e.m., n=9 mice. **(c)** Individual representative example depicting the change in cortical neuronal activity as a function of voluntary wheel running. Top: multiunit activity (MUA) trace recorded from M1 together with corresponding RW-activity (bottom, each bar represents a single wheel rung count) in the same animal. Corresponding waveforms of the action potentials recorded extracellularly from these channels are shown on the right. **(d)** Average cortical firing rates in M1 shown as a function of running speed (x-axis). Thick lines: mean values, s.e.m., n=9 mice. Values from individual animals are shown as thin line plots. **(e)** Two representative examples of cortical local field potentials (LFP) in M1 during high speed running during sleep deprivation. Top: LFPs recorded concomitantly in three channels of the 16-channel microwire array. Corresponding MUA is shown below as a raster (each bar is an individual spike). Bottom: corresponding RW-activity. Note that positive LFP waves (arrows) are accompanied with reduced MUA.

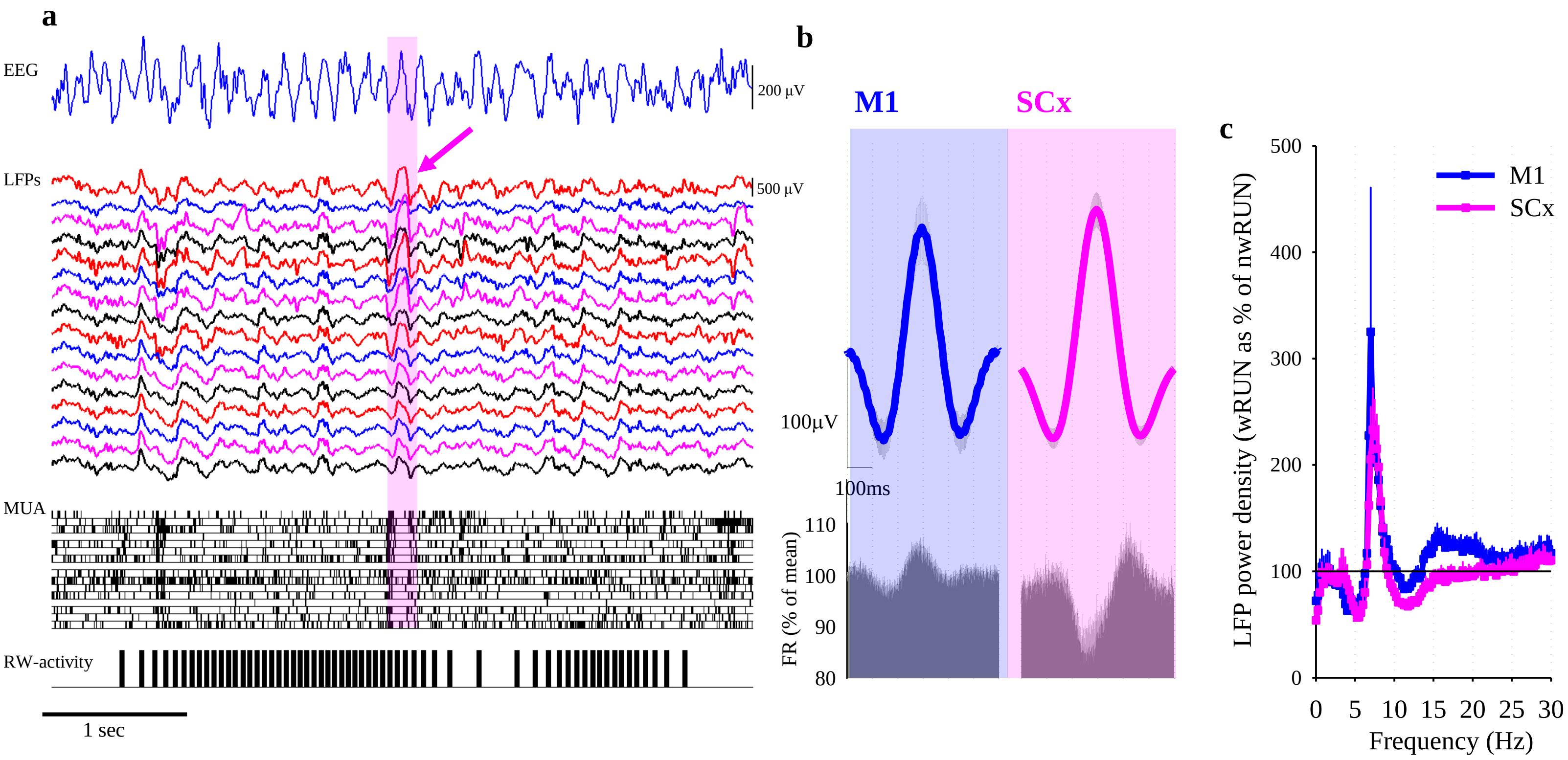

Supplementary Figure 10. Cortical activity during voluntary wheel running. (a) Representative example of EEG, LFP and MUA in the somatosensory cortex (SCx) during high speed running. Top: EEG recorded in the occipital derivation. Note the presence of strong EEG theta-activity, typical for running. Traces below represent local field potentials (LFP) recorded concomitantly in all sixteen channels of the 16-channel microwire array. Corresponding multiunit activity (MUA) is shown below as a raster (each bar is an individual spike). Bottom: corresponding running-wheel (RW)-activity (each bar represents a single wheel rung count). Note that a positive LFP wave (arrow) is accompanied with reduced MUA across most of the 16 channels. **(b)** Average LFP slow (2-6 Hz) wave recorded during waking, and corresponding average MUA from all recording channels in the primary motor cortex (M1) and SCx. Mean values, n=7 mice (M1) and n=5 mice (SCx), s.e.m.. Note that in both regions MUA during waking is modulated by LFP slow waves, but only a moderate reduction of firing rates is observed in the SCx in association with positive LFP wave. **(c)** LFP spectral power density calculated for signals recorded in M1 and SCx. The spectra during running waking (wRUN) are shown as percentage of corresponding spectra during non-running (nwRUN) waking. Mean values, s.e.m. (n=7 and 5 mice for M1 and SCx recordings respectively).

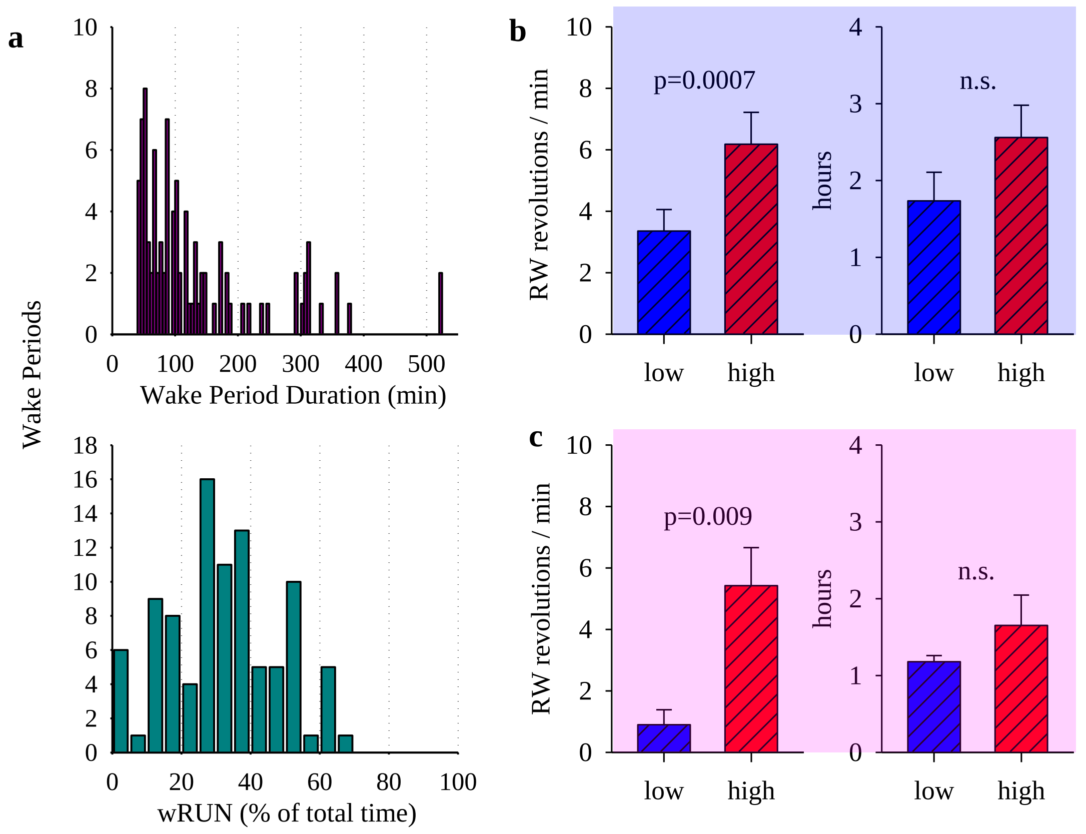

Supplementary Figure 11. Characteristics of running wheel activity and spontaneous waking periods. (a) Top: Distribution of all spontaneous waking periods > 40 min in n=16 mice pooled from both experiments, where primary motor cortex (M1) and somatosensory cortex (SCx) recordings were performed (95 waking periods in total), as a function of their duration. Bottom: distribution of all waking periods as a function of time spent running (wRUN) during corresponding periods. **(b,c)** Left: all waking periods > 40 min across all animals were subdivided into two 50% percentiles based on the amount of running wheel (RW) activity, which resulted in ~ 2-3-times difference in the amount of running between 'low' and 'high' running waking periods. Right: Corresponding duration of waking periods categorised based on the proportion of running-wheel activity. Mean values, s.e.m., top: n=9 mice implanted in M1, bottom: n=5 mice implanted in SCx.

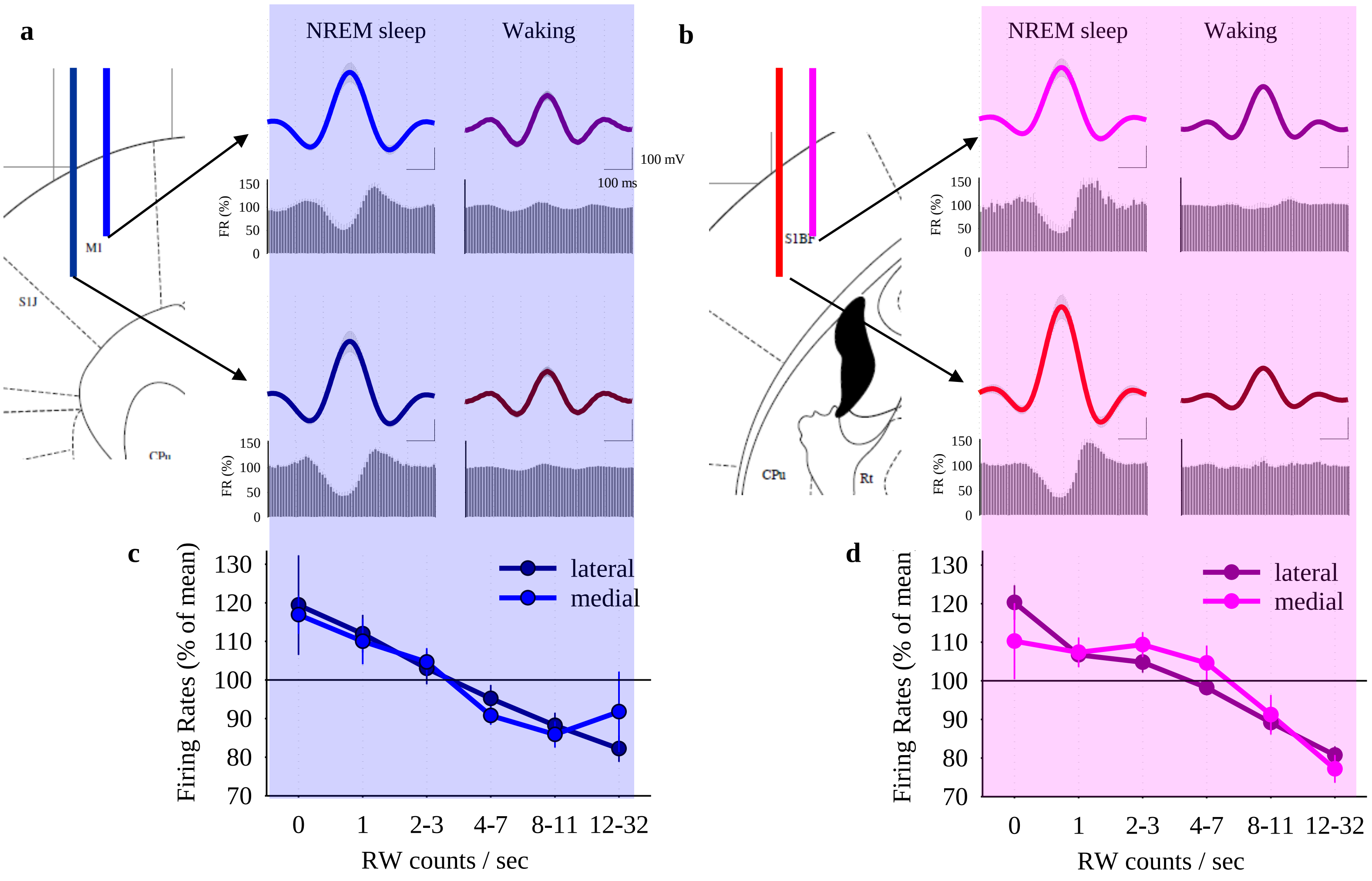

Supplementary Figure 12. The effects of electrode depth on LFP and MUA and on the relationship between firing rates and running speed. **(a)** Left: Schematic depiction of the relative position of the longer and shorter rows of wires on the 16-ch microwire array within M1. Right: Average local field potential (LFP) slow wave recorded during NREM sleep (0.5-4 Hz) and waking (2-6 Hz), and corresponding average MUA recorded across all recording channels shown separately for the longer and shorter row of the array. Mean values, s.e.m., n=7 mice. **(b)** Same for the somatosensory cortex (SCx), n=5 mice. **(c)** Firing Rates (FR) in M1 shown as a function of running speed. The curves show separately firing rates calculated for putative neurons recorded with the longer and shorter row of wires. Mean values, s.e.m., n=9 mice. **(d)** The same as (**c**) for SCx. Mean values, s.e.m., n=5 mice. Note that overall the position of the recording electrodes within M1 and SCx has minor influence on both the relationship between LFP and corresponding MUA and change in firing rates as a function of running speed .

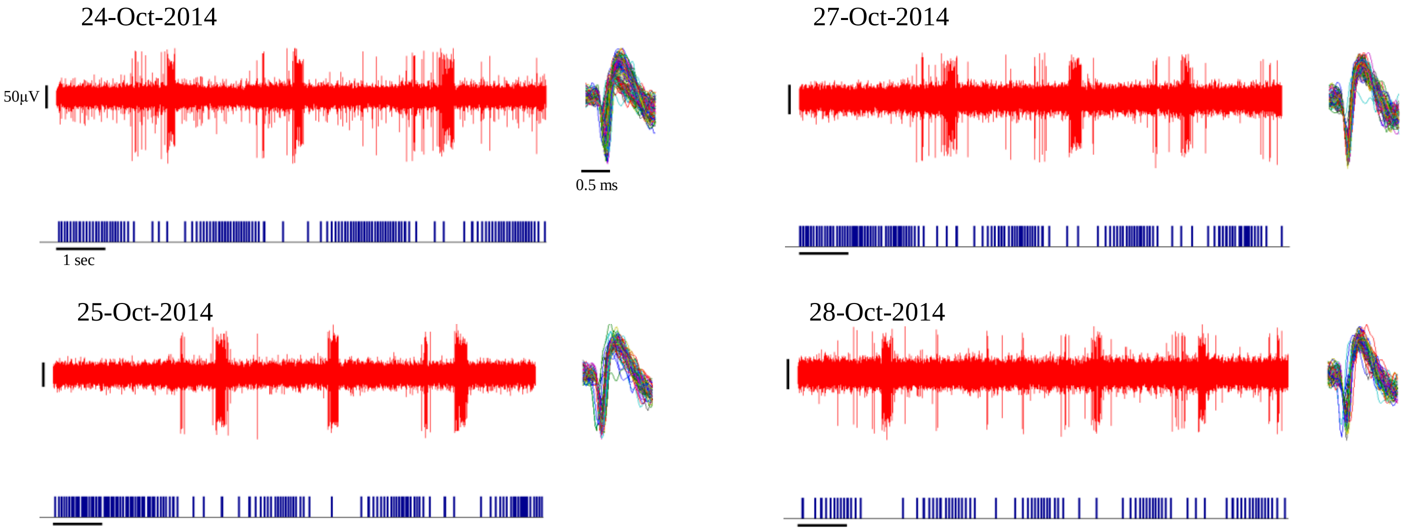

Supplementary Figure. 13. Representative example of a putative single unit recorded in M1, which shows a stable relationship with running wheel activity across several consecutive days. Note that at least two neurons can be detected in the trace recorded on 24-Oct-2014, but the putative single unit with a higher spike amplitude can also be detected on subsequent days. This unit has a similar spike wave shape across days and consistently fires mostly during brief cessations in running.

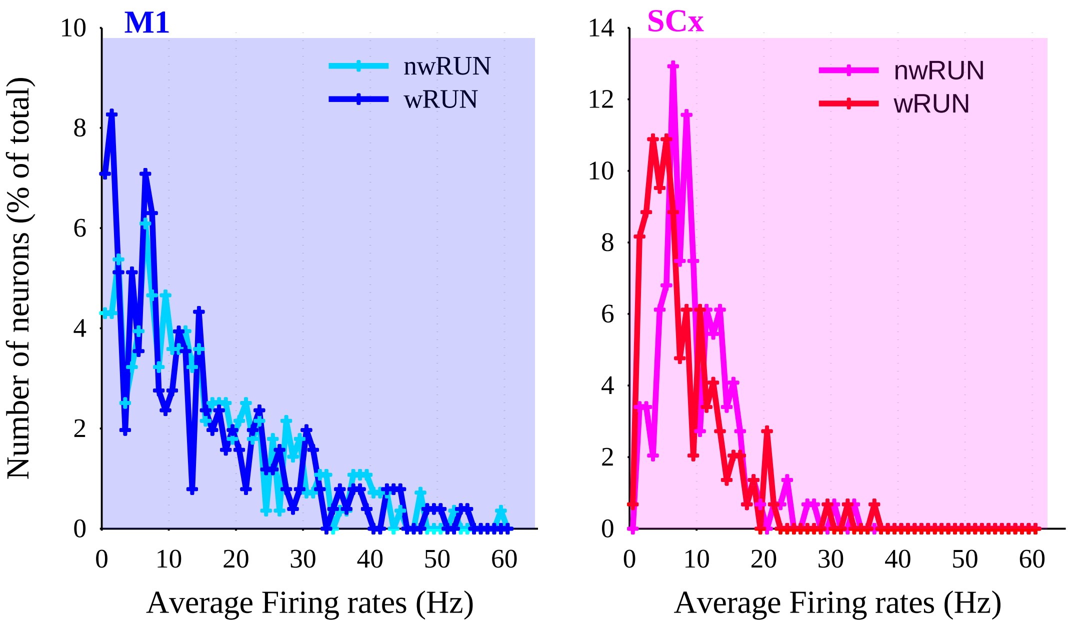

Supplementary Figure 14. Distribution of individual putative single units as a function of their spontaneous average firing rates during nwRUN waking and RUN waking (M1 and SCx: 279 and 147 putative single units in n=9 and n=5 mice respectively). Note that most putative single units have relatively slow firing rates and only a small proportion have firing rates > ~ 15 Hz.
